# SonoVue^®^ vs. Sonazoid™ vs. Optison™: Which Bubble Is Best for Low-Intensity Sonoporation of Pancreatic Ductal Adenocarcinoma?

**DOI:** 10.3390/pharmaceutics14010098

**Published:** 2022-01-01

**Authors:** Spiros Kotopoulis, Mihaela Popa, Mireia Mayoral Safont, Elisa Murvold, Ragnhild Haugse, Anika Langer, Georg Dimcevski, Christina Lam, Tormod Bjånes, Odd Helge Gilja, Emmet Mc Cormack

**Affiliations:** 1 National Centre for Ultrasound in Gastroenterology, Haukeland University Hospital, Jonas Lies vei 65, 5021 Bergen, Norway; ethode12@hotmail.com (E.M.); odd.helge.gilja@helse-bergen.no (O.H.G.); 2 Department of Clinical Medicine, University of Bergen, Jonas Lies vei 65, 5021 Bergen, Norway; georg.dimcevski@gmail.com; 3 Neoety AS, Borgenhagen 109, 2040 Kløfta, Norway; 4 KinN Therapeutics AS, Jonas Lies vei 65, 5021 Bergen, Norway; mihaela.popa@uib.no (M.P.); mireia.safont@uib.no (M.M.S.); 5 Centre for Cancer Biomarkers CCBIO, Department of Clinical Science, University of Bergen, Jonas Lies vei 65, 5021 Bergen, Norway; ani-lan@web.de (A.L.); emmet.mc.cormack@uib.no (E.M.C.); 6 Centre for Pharmacy, Department of Clinical Science, University of Bergen, Jonas Lies vei 65, 5021 Bergen, Norway; ragnhild.haugse@uib.no (R.H.); christina.lam9@gmail.com (C.L.); 7 Department of Quality and Development, Hospital Pharmacies Enterprise in Western Norway, Møllendalsbakken 9, 5021 Bergen, Norway; 8 Department of Medical Biochemistry and Pharmacology, Section of Clinical Pharmacology, Haukeland University Hospital, Jonas Lies vei 65, 5021 Bergen, Norway; tormod.karlsen.bjanes@helse-bergen.no; 9 Department of Internal Medicine, Haematology Section, Haukeland Haukeland University Hospital, Jonas Lies vei 65, 5021 Bergen, Norway

**Keywords:** ultrasound, pancreatic cancer, sonoporation, microbubbles, ultrasound-enhanced therapy, targeted drug delivery

## Abstract

The use of ultrasound and microbubbles to enhance therapeutic efficacy (sonoporation) has shown great promise in cancer therapy from in vitro to ongoing clinical studies. The fastest bench-to-bedside translation involves the use of ultrasound contrast agents (microbubbles) and clinical diagnostic scanners. Despite substantial research in this field, it is currently not known which of these microbubbles result in the greatest enhancement of therapy within the applied conditions. Three microbubble formulations—SonoVue^®^, Sonazoid™, and Optison™—were physiochemically and acoustically characterized. The microbubble response to the ultrasound pulses used in vivo was simulated via a Rayleigh–Plesset type equation. The three formulations were compared in vitro for permeabilization efficacy in three different pancreatic cancer cell lines, and in vivo, using an orthotopic pancreatic cancer (PDAC) murine model. The mice were treated using one of the three formulations exposed to ultrasound from a GE Logiq E9 and C1-5 ultrasound transducer. Characterisation of the microbubbles showed a rapid degradation in concentration, shape, and/or size for both SonoVue^®^ and Optison™ within 30 min of reconstitution/opening. Sonazoid™ showed no degradation after 1 h. Attenuation measurements indicated that SonoVue^®^ was the softest bubble followed by Sonazoid™ then Optison™. Sonazoid™ emitted nonlinear ultrasound at the lowest MIs followed by Optison™, then SonoVue^®^. Simulations indicated that SonoVue^®^ would be the most effective bubble using the evaluated ultrasound conditions. This was verified in the pre-clinical PDAC model demonstrated by improved survival and largest tumor growth inhibition. In vitro results indicated that the best microbubble formulation depends on the ultrasound parameters and concentration used, with SonoVue^®^ being best at lower intensities and Sonazoid™ at higher intensities.

## 1. Introduction

Sonoporation was initially defined as the transient formation and resealing of pores in a cell membrane due to combined microbubble and ultrasound (US) treatment, which may be used therapeutically to increase uptake and entrapment of drugs or genes in cells [1,2]. Sonoporation is particularly promising for enhancing the treatment of cancer, a leading cause of morbidity and mortality worldwide. The most common drug treatment offered to cancer patients is chemotherapy, which is limited by toxicity and development of drug resistance that often result in therapy failure. 

To have a therapeutic effect on the target disease a sufficient amount of the drugs must reach their cellular target. Even when drugs are administered intravascularly, tumoral drug delivery is hindered by numerous biological barriers such as the endothelium of the blood vessels, the heterogeneous and disorganized tumor blood vessels, disrupted blood supply, the extracellular matrix (ECM), the stroma and microenvironment of the tumor, high interstitial and osmotic pressure, and limited ability to cross the cell membrane of the cancer cells [3,4]. Many cytotoxic agents, despite effectively killing cancer cells in vitro, have insufficient effect on cancer cell viability in vivo [5]. Most of the research done to solve the drug delivery problem focuses on the development of drug carriers like liposomes and nanoparticles, taking advantage of passive enhanced permeability and retention effect (EPR-effect) [6,7,8]. Though the main advantage is reduction of side effects instead of the improvement of overall survival compared to free drug [6,7], this kind of targeted drug delivery has still not fully met the expectations, as the enhancement of drug delivery by passively relying on the EPR effect is too limited and heterogenous [7,8]. Therefore, it has been suggested to use vessel-modulating strategies, including physical methods such as sonoporation, to improve efficiency and specificity of the EPR effect [7]. 

In fact, the use of microbubbles and low-intensity ultrasound to increase efficacy of chemotherapy has been demonstrated to reduce tumor growth in a range of preclinical trials of different cancer types [9,10,11,12,13,14]. Therefore, sonoporation of pancreatic ductal adenocarcinoma (PDAC) has gained increased attention. PDAC is a highly deadly cancer, characterized by pronounced desmoplasia, a complex, hypoxic tumor microenvironment and resistance to chemotherapy [15]. The combination of gemcitabine and commercial microbubbles (SonoVue^®^) [16], paclitaxel in combination with the Acoustic Cluster Therapy (ACT^®^) concept [17], and Nab-paclitaxel (nanoparticle formulation of paclitaxel) combined with microbubbles [18] improved PDAC therapy. In 2016, results from the first Phase I human clinical trial using ultrasound and microbubbles in combination with chemotherapy were published [19]. The study demonstrated safety and further indicated prolonged survival of patients diagnosed with PDAC and treated with sonoporation with chemotherapy (gemcitabine). Currently, additional sonoporation clinical trials have been initiated in breast, colorectal cancer, and liver metastases [20,21,22,23] using ultrasound parameters in the approved diagnostic range. While other studies have been initiated to enhance delivery across the blood–brain barrier (BBB) to treat glioblastoma [24,25,26] using ultrasound parameters higher the diagnostic range.

The use of commercially available microbubbles and ultrasound equipment approved for use in diagnostic ultrasound imaging together with co-administration of systemic chemotherapy represent the fastest bench-to-bedside translation of sonoporation. In the first Phase I clinical trial [19] choice and dose of microbubbles, and choice of ultrasound parameters was limited to parameters approved for diagnostic ultrasound imaging, but still the study indicates that sonoporation using these parameters can significantly prolong survival. However, many factors influence the outcome of sonoporation efficacy and there is currently no consensus on the optimal combination of ultrasound parameters, choice of microbubble formulation or microbubble concentration for best sonoporation efficacy. To provide a better understanding, we have investigated the impact of sonoporation on the in vitro uptake of molecules, over a range of microbubble concentrations and ultrasound intensities, and on the in vivo efficacy of sonoporation and paclitaxel (PTX). Three microbubble formulations were evaluated: SonoVue^®^ (distearoylphosphatidylcholine and macrogol 4000 stabilized sulfur hexafluoride), Sonazoid™ (hydrogenated egg phosphatidyl serine stabilized perflubutane), and Optison™ (human albumin-stabilized octafluoropropane). These microbubble formulations were chosen as they are currently the only three approved agents for clinical diagnostic use in Norway.

Simulations and acoustic characteristics were also performed in an attempt to decipher which bubble response parameters may be of interest in determining effective sonoporation. A definite decision of which are the best microbubble and ultrasound parameters cannot be determined, as these are highly interdependent. The preclinical study showed that sonoporation can enhance cancer therapy even at a very low intensity, and at this ultrasound intensity SonoVue^®^ provided the largest therapeutic enhancement.

## 2. Materials and Methods

### 2.1. Microbubble Characterization

#### 2.1.1. Reconstitution of Microbubbles

Microbubbles were aspirated via a 19 G needle, and a 19 G venting needle was used to avoid a pressure drop in the vial. Sonazoid™ (GE Healthcare, Little Chalfont, UK) was reconstituted by adding 2 mL NaCl 9 mg/mL (B Braun, Melsungen, Germany) with a venting needle and gently agitated for 30 s. SonoVue^®^ (Bracco S.p.A., Milan, Italy) was reconstituted in accordance with manufacturer’s specifications. Optison™ (GE Healthcare) microbubbles were gently agitated until no residue was visible at the base of the vial.

#### 2.1.2. Size, Concentration and in Vial Stability

To ensure an equitable comparison between all three microbubble agents, it is essential that as many parameters as possible are controlled. Due to the different formulations and preparation methods, it is expected that each agent has a different size and stability. Size, concentration, and stability were evaluated using optical microscopy to confirm literature and manufacturer values. Immediately after preparing a vial, a sample (100–200 µL) was removed and diluted 1:100 to 1:500 in saline (Fresenius Kabi, Bad Homburg vor der Höne, Germany). This dilution was necessary to be able to visualise and measure single bubbles instead of agglomerates. A 25 µL sample of the diluted bubbles was placed in the central aperture of a 100 µm thick spacer on a glass slide and covered with size 0 thickness glass coverslip. Images were captured using an upright microscope (Nikon Eclipse E200, Nikon Instruments Europe B.V., Amsterdam, The Netherlands) and an ELWD 40x/0.60NA CFI S Plan Fluor objective (Nikon Instruments Europe B.V) via an Infinity 1-3 camera (Lumenera Corporation, Ottawa, ON, Canada). When required, a focused stacked image was captured where each image in the focus stack was spaced by 1 µm (the objective depth of field was approximately 2 µm) until all bubbles in the field of view were in focus (10–25 images). Three to five images were taken from each sample followed by a new sample from the same dilution. Every 2–5 min a new sample was taken from the vial and the process was repeated for a total duration of 1 h. A total of 3 vials were evaluated from each microbubble agent. The focus stacks were converted into a single 2D image using Helicon Focus (HeliconSoft, Kharkiv, Ukraine) using the in software labelled “method B” with a radius of 20, to reduce the halo artifact, and smoothing of 3. The resultant images were then processed in MIPAR (MIPAR, Worthington, OH, USA) to determine the diameter and particle count. Following single bubble detection and segmentation, the diameter of each bubble was measured using the “equivalent diameter” feature which calculates the diameter of each particle if each was a circle of the equivalent area of the detected particle. Supplemental Figure S1 depicts a schematic on how the parameters were calculated. A minimum bubble diameter cut-off was set at 0.7 µm, as this was 30% larger than the objective resolution (meaning that each detected bubble had to be a minimum of 5 pixels in diameter). Roundness was calculated as a ratio of the equivalent diameter to the maximum diameter. The reported geometric mean values were calculated from all measurements made within the first 1 min or 30 ± 0.5 min after preparing the microbubbles. The concentration was calculated by multiplying the total number of bubbles in the field of view by the imaging volume of 6.2 × 10^−^^6^ mL. 

#### 2.1.3. Attenuation Measurements

**Figure 1:**
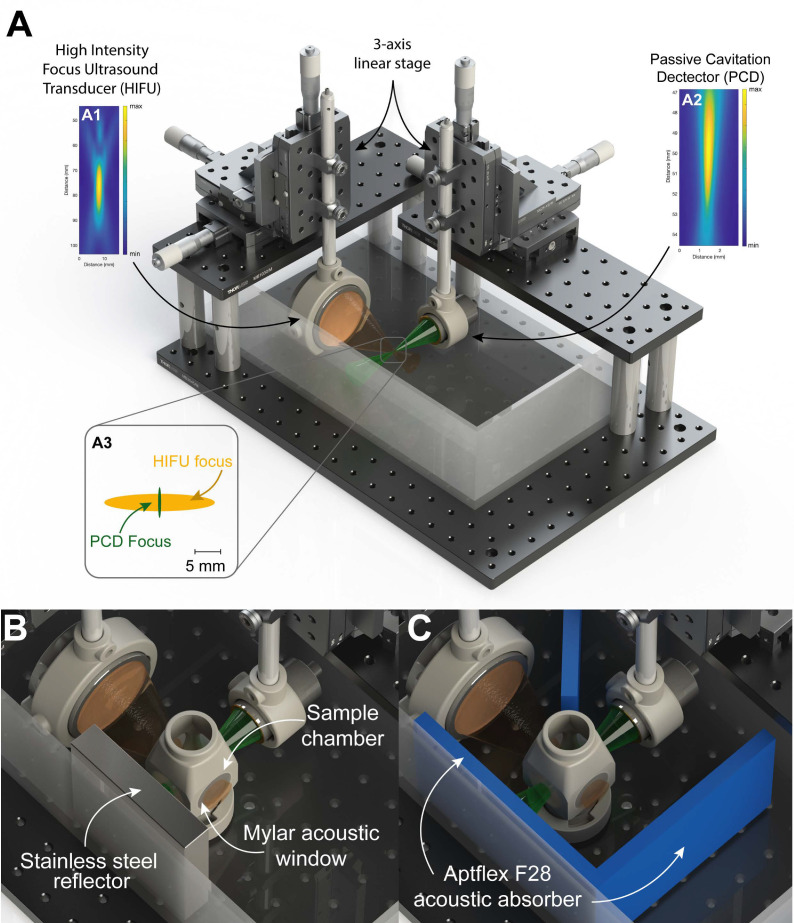
Graphical rendering of the experimental setup showing how the microbubble attenuation and cavitation thresholds were measured. (**A**) shows an overview of the ultrasound transducers with their corresponding focal sizes and alignment. (**B**) shows how the attenuation was measured using only the PCD and a stainless-steel reflector. (**C**) shows how the cavitation thresholds were measured using the confocally arranged transducers.

The bulk resonance frequency was determined by measuring the acoustic attenuation as a function of frequency. The bulk resonance frequency was assumed to be the frequency with the highest attenuation. Figure 1 shows a graphical rendering of the experimental setup used. Figure 1A shows a general overview of experimental configurations without the sample chamber. Two transducers were used: a single element, lead zirconate titanate, high-intensity focused ultrasound (HIFU), 65 mm Ø, focused, ultrasound transducer with a focal depth of 75 mm (Figure 1(A1)) (Precision Acoustics Ltd., Dorset, Dorchester, UK) and a polyvinylidene fluoride (PVDF), 23 mm Ø, passive cavitation detector (PCD), with a focal depth of 49.5 mm (Figure 1(A2)) (Precision Acoustics Ltd., Dorset, Dorchester, UK). Both transducers were calibrated in a water tank using a 200 µm needle hydrophone (Precision Acoustics Ltd., Dorset, Dorchester, UK). The transducers were arranged perpendicularly to each other, and the acoustic foci were aligned by moving the transducers in 3-axis and finding the maximal echo of a 1.5 mm spherical point reflector using a pulser/receiver (5072PR, Olympus Scientific Solutions, Waltham MA, USA) connected to a 200 MHz oscilloscope (DSOX3024A, Keysight Technologies, Santa Rosa, CA, USA). The HIFU transducer was aligned using impedance matching circuit of 3.28 MHz to improve spatial accuracy. Supplemental Figure S2 shows the acoustic output waveforms and the frequency spectra of the transducers when driven from the pulser/receiver for the PVDF transducer and radio frequency (RF) amplifier (2200L, Engineering and Innovation LTD, Rochester, NY, USA) via a waveform generator (33500B, Keysight) with a 20-cycle pulse for the HIFU transducer. The −3dB focal volume was 125 mm^3^ for the HIFU transducer at 1.08 MHz, and 11 mm^3^ for the PVDF transducer.

The sample chamber was 3D printed using a FLGPWH03 resin (Formlabs, Somerville, MA, USA) with 32 mm Ø windows for acoustic propagation. The acoustic windows were sealed using 23 µm thick mylar sheets and glued on using the same FLGPWH03 resin and cured in an ultraviolet (UV) oven. The total used volume of the sample chamber was 80 mL.

The experimental configuration shown in Figure 1B was used to measure the microbubble induced acoustic attenuation. In this configuration only the PVDF transducer was used. A 2 cm thick stainless-steel block was used as the reflector and placed approximately 8 cm from the transducer face. The pulser/receiver was set at a pulse repletion frequency (PRF) of 100 Hz, an energy level of 2, damping of 50 Ω, and a gain of 40 dB. The water bath was warmed to a temperature of 37 °C. The sample chamber was filled with saline supplemented with 5% bovine serum albumin (Merck KGaA, Darmstadt, Germany) which was warmed to 44 °C for 24 h and reduced to 37 °C for 4 h prior to use to attempt matching typical blood gas saturation. Twenty baseline waveforms (without microbubbles) were recorded for background subtraction. Bubbles were removed from the vial (as described in Section 2.1.1) and added to the sample chamber and the fluid was gently agitated using a 1 mL pipette six times before each measurement. For each concentration 15 recordings (1 s apart) were captured during gentle agitation with a 1 mL pipette placed outside the sound field at the edge of the container. Thirteen concentrations were evaluated (0.2 × 10^5^–7 × 10^5^ ppmL) over a 5 min period. At the highest concentration, almost no ultrasound could pass through the sample. All concentrations were repeated three times.

The acoustic attenuation spectra were calculated in MATLAB by obtaining the power spectrum (pspectrum function in MATLAB) for all individual waveforms, calculating the mean of the replicated measurements, and subtracting the averaged baseline from the averaged measurements with bubbles. The attenuation was converted to dB/cm by dividing the attenuation via the sample chamber thickness ×2; a total of 8 cm.

#### 2.1.4. Stable to Inertial Cavitation Transition

The same experimental setup as described in Section 2.1.3 (Attenuation Measurements) was used to determine an “inertial” cavitation threshold. The experiment was performed as shown in Figure 1C. Acoustic absorbing material (Aptflex F28, Precision Acoustics Ltd., Dorchester, UK) was placed at the tank walls to minimize echoes and in between the two transducers to minimize potential cross-talk.

The cavitation measurements were performed at a microbubble concentration of 2 × 10^5^ ppmL. This concentration was chosen as it would match a feasible clinical scenario, i.e., one vial of SonoVue^®^ (5 mL), 47% of an Optison™ vial (1.4 mL), and 42% of a Sonazoid™ vial (0.83 mL). The −3dB intersecting area between the acoustic foci was estimated to be 0.2–0.3 mm^3^, meaning approximately 50 microbubbles were evaluated with each measurement.

The PZT ultrasound transducer applied ultrasound at 26 different mechanical indexes (MI’s) from 0.01 to 2.0, with and without microbubbles, whilst the sample was continuously agitated. Each MI was sequentially repeated 15 times. The experiment was repeated 3 times, i.e., 90 waveforms per MI. The power spectrum was calculated for each recorded waveform at the same MI and averaged. The averaged power spectrum without bubbles was subtracted from the averaged spectrums with bubbles at the same MIs. Two parameters were evaluated to determine the inertial cavitation index: (1) The subharmonic magnitude (between 400–600 kHz) [27] and the spectral integral [28] (from 200 kHz to 10 MHz). A four-point logistic curve (Equation (1)).

(1) $$Y=d+\frac{\left(a-d\right)}{1+{\left(\frac{x}{c}\right)}^{b}}$$

where a and d are the upper and lower plateaus respectively, c is the point of inflection and b Hill’s slope was fit to the data to determine a comparable cavitation threshold. For all datasets a and d were set to equal values. The MI at the point of inflection (c) was chosen as the representative “cavitation threshold”.

### 2.2. Simulations

All simulations were performed in MATLAB R2018B (Mathworks, Natick, MA, USA).

#### 2.2.1. Microbubble Pharmacokinetics

To estimate and correlate in vivo and in vitro concentrations, microbubble pharmacokinetics were simulated using conventional equations [29]. Specifically, the plasma concentration of the microbubbles (*C*) during infusion was estimated using

(2) $$C=\left(\frac{k_{0}}{CL}\cdot \left(1-e^{-k_{e}\cdot t}\right)\right)\cdot C_{bub}$$

where $k_{0}\;$is the infusion rate (20 µL/min); $CL=k_{e}\cdot V_{d}$ is the clearance rate; $k_{e}=0.6931/t_{1/2}$ is the elimination rate; $t_{1/2}$ is the bubble half-life; $V_{d}$ is the volume of distribution, i.e., the mouse blood volume (2.5 mL); and $C_{bub}$ is the original microbubble concentration in particles per mL (ppmL).

As microbubble half-life varies depending on formulation, half-lives from 30–180 s were simulated.

#### 2.2.2. Simulated Microbubble Behavior

A modified version of the Rayleigh–Plesset equation (Equation (3)) [30,31] was solved using the ODE45 function in MATLAB to attempt to predict which behavior was important for low intensity sonoporation. Specifically, the equation was

(3) $$\ddot{R}=\frac{1}{\rho R}\left[-\frac{3}{2}\rho {\dot{R}}^{2}+p_{g}{\left(\frac{R_{0}}{R}\right)}^{3\gamma }+p_{v}-\frac{2\sigma }{R}-2\chi \left(\frac{1}{R_{0}}-\frac{1}{R}\right)-\delta_{t}\omega \rho R\dot{R}-p_{ac}\left(t\right)\right]$$

where *R* is the instantaneous bubble radius, *ρ* is the fluid density, $p_{g}$ is the initial gas pressure, $p_{v}$ is the vapour pressure, *γ* is the specific heat ratio, σ is the interfacial tension, χ is the shell stiffness, *δ_t_* is the damping coefficient [32], *ω* is the frequency in radians, and $p_{ac}\left(t\right)$ is the acoustic pressure at a given time. The radiated acoustic pressure on the cells was determined by calculating the peak radiated pressure 7 µm from the microbubble centre. This distance was chosen as the typical diameter of a blood vessel in PDAC is 10–20 µm [33]; thus, this would mean the microbubble is approximately in the middle of the capillary, i.e., a worst-case scenario. A schematic illustration is shown in Supplemental Figure S3.

The microbubble properties were directly extracted from literature values or calculated from literature values (Table 1). Mouse blood fluid properties were used as surrounding fluid. Two ultrasound pulses (as used in the preclinical experiments) were evaluated, a short B-mode pulse, and a long 20 µs “treatment” pulse (see Section 2.4.1 for more details). The ultrasound pulses were recorded in a water tank using a 200 µm diameter PVDF needle hydrophone (Precision Acoustics Ltd.) and converted to pressured using frequency deconvolution. The ultrasound pulses were generated from a GE Logiq E9 ultrasound system coupled to a C1-5 ultrasound probe. R_0_ values from 0.35 to 10 µm in steps of 50 nm were simulated.

### 2.3. In Vitro Cell Experiments

#### 2.3.1. Chemicals

All chemicals were purchased from Merck KGaA (Darmstadt, Germany) unless otherwise stated.

#### 2.3.2. Cell Culture

Three types of PDAC cell lines—MIA PaCa-2, PANC-1, and BxPC-3—were kindly donated from Professor Anders Molven (University of Bergen, Norway). MIA PaCa-2 cells were cultured in high-glucose Dulbecco’s Modified Eagle’s Medium (DMEM) supplemented with 10% fetal bovine serum (FBS), 2% L-glutamine, 1 mM sodium pyruvate, and 2.5% horse serum. PANC-1 cells were cultured in high-glucose Dulbecco’s Modified Eagle’s Medium (DMEM) supplemented with 10% FBS, 1% L-glutamine and 1 mM sodium pyruvate. BxPC-3 cells were cultured in RPMI 1640 medium supplemented with 10% FBS, 2% L-glutamine and 1 mM sodium pyruvate. All cells were cultured in a 5% CO_2_ humidified atmosphere at 37 °C. 

Between 2–3 days prior to experiments, 26 mL of cell suspension was injected in Petaka G3 LOT^®^ (Low Oxygen Transport) cell culture chambers (Celartia Ltd., Powell, OH, USA). The Petaka G3 LOT^®^ chambers (subsequently called Petaka’s) allow for cell culture in a hypoxic environment, mimicking the hypoxic nature of a PDAC tumor. A suitable number of cells was injected based on growth curves [48], and cells were treated with ultrasound at 70–80% confluency. The air valve was sealed with tape to avoid influx of gas, and the cell culture chambers were oriented in horizontal position for minimum 24 h to allow for cells to adhere to the plastic surface. After 24–48 h the Petakas were oriented vertically, with the injection port, and air valve placed uppermost.

#### 2.3.3. Microbubble Dilution

Small volumes of microbubbles were withdrawn from the vials, transferred to microcentrifuge tubes, and further diluted in NaCl 9 mg/mL (B Braun or Fresenius Kabi) immediately prior to injection into Petaka’s to ensure accurate microbubble concentrations. Four concentrations of microbubbles were used during treatment: 0.28 × 10^6^, 2.8 × 10^6^, 4.6 × 10^6^, and 6.5 × 10^6^ ppmL. Sonazoid™ bubbles were used within 1 h of reconstitution, SonoVue^®^ within 30 min of reconstitution and Optison™ within 20 min of perforation of the rubber stopper of the vial.

#### 2.3.4. In Vitro Sonoporation

Ultrasound was applied via a “plate sonicator” described in previous publications (Supplemental Video S1) [48,49]. The ultrasound transducer consisted of 128, 9 × 6 × 2 mm, PZ26 elements spaced in an 8 × 16 grid with a 1 mm spacing between elements. The plate sonicator was driven by a 128-channel open ultrasound system (Lecoeur Electronique, Chulles, France). The ultrasound system was multiplexed in eight groups of 16 channels. Each group fired a plane wave of the required ultrasound parameters 28 times, at a pulse repetition time of 200 µs, then immediately switched to the next group of elements. This resulted in an overall pulse repetition frequency of 22 Hz.

A non-toxic, cell impermeable dye, calcein, was added during sonication. A 50 mg/mL calcein stock solution in 1 M NaOH was kept at 2−8 °C protected from light. Immediately prior to sonication, calcein (6 μM) ± microbubbles were diluted in NaCl 9 mg/mL to a total volume of 1 mL and injected into the Petaka. Subsequently, the Petaka was gently rolled in all directions to ensure homogenous distribution of the microbubbles and calcein. Any air pockets/bubbles were removed from the Petaka via the injection port following manufactures guidelines before ultrasound treatment.

Each Petaka was placed in the carriage frame and lowered into the plate sonicator water bath with the cells on the upper culture surface to ensure that the floating microbubbles would contact the cells. Before ultrasound treatment, a low MI (<0.01) B-mode scan was performed using the same device to ensure that no air pockets were trapped between the Petaka and the water interface. Three different ultrasound conditions were used (Table 2), referred to as “Low”, “Medium”, or “High”. Treatment ultrasound was applied for 5 min. The control samples were placed in the plate sonicator for 5 min without application of ultrasound. After sonication the cells were incubated for 1 h (from the start of ultrasound exposure) to allow cell membrane pores to reseal [50].

Three independent replicates were performed for each condition in random order and replicates were sampled three times.

#### 2.3.5. Flow Cytometry

After incubation, the cell culture medium was removed from the Petaka and cells were flushed twice with phosphate buffered saline (PBS). Cells were detached from the Petaka using 0.25% trypsin. Cell culture medium was added to neutralize the trypsin and the cell suspension was harvested and transferred to flow cytometry tubes. The cell suspension was centrifuged, resuspended in PBS and filtered through a 40 μm cell strainer (Avantor, Radnor; PA, USA) before flow cytometric analysis. Cells were analyzed using an Accuri C6 flow cytometer (BD Bioscience, Franklin Lakes, NJ, USA). Data collected from the Acurri C6 were gated in FlowJo^®^ (v10.6, BD Bioscience), and uptake of calcein was measured as percentage calcein-positive cells. The gating strategy is shown in Supplemental Figure S4. In summary, single cells were selected by manually identifying the primary population in a forward scatter area vs. height plot. A manual polygon was used to select the population. This selected population was then viewed in a fluorescence area at 533/30 nm vs. side scatter area plot. A rectangular gate was manually placed to the right of the primary population ensuring that <1% of the population was selected. This same gate was then placed over the single cell population of the same experimental batch to determine the number of calcein-positive cells.

### 2.4. Pre-Clinical Mouse Experiments

All animal experiments were approved by The Norwegian Animal Research Authority (Application number: 15/251788, approved 15 February 2016) and conducted according to The European Convention for the Protection of Vertebrates Used for Scientific Purposes. During depilation and imaging, mice were anaesthetized with 2% isoflurane (Isoflo^®^ vet, Orion Pharma, Norway) in oxygen. During treatment, animals were anesthetized in 2% isoflurane in medical air, and sustained under anesthesia between 1 and 1.5% isoflurane.

A previously published orthotopic xenograft model of MIA PaCa-2^luc^ cells in NOD-scid IL2rγ^null^ mice (Vivarium, University of Bergen; originally a generous gift of Prof. Leonard D. Shultz, Jackson Laboratories, Bar Harbour, ME, USA) was used [16]. In brief, this model was developed by exteriorizing the pancreas and injecting 10^6^ MIA PaCa-2^luc^ cells suspended in 20 µL of 25/75 *v*/*v* Matrix Matrigel (Corning, NY, USA) and phosphate-buffered saline solution, using a 30-gauge needle.

#### 2.4.1. Optical Imaging

Mice were imaged once a week, two days after ultrasound treatment, using an In-Vivo FX PRO molecular imaging system (Carestream Health, Inc., Rochester, NY, USA). The mice were anesthetized using isoflurane in O_2_ and 150 mg/kg D-luciferin (Biosynth AG, Staad, Switzerland) was injected intraperitoneally (IP) 10 min prior to imaging. The left flank of the mice was imaged to ensure the complete tumor was captured in a single scan. Total bioluminescence values were measured using manual ROIs with background correction in the Carestream MI software (Standard Edition, v.5.0.6.20, Carestream Health, Inc.). A whole-body ROI was used to include metastatic spread.

#### 2.4.2. Ultrasound Imaging

Once a significant signal was detected from the bioluminescence imaging (i.e., week 2), ultrasound imaging followed by treatment was performed weekly. The mice were anesthetized and kept under anesthesia using isoflurane in air. The mice were placed on a heating pad set to 37 °C in dorsal recumbency and abdominal hair was removed using depilatory cream (Veet Sensitive, Reckitt Benckiser, Slough, UK). Three-dimensional images of the primary tumor were captured using a Vevo 2100 ultrasound scanner combined with a MS400 (18−38 MHz) probe and 1D stage (VisualSonics Inc., Toronto, ON, Canada). The “Enhanced abdominal imaging B-mode” preset, and 3D Power Doppler in combination with B-mode was used. A custom Power Doppler preset was made maximizing the sensitivity (based on “Very Slow Flow”) of the system with minimal “wall” artifacts. The settings were briefly optimized for each mouse to achieve a balance between minimizing artifacts and maximizing sensitivity. Color Doppler imaging was also used to aid primary tumor identification. Respiration gating was used during 3D image acquisition. An additional heat-lamp ensured optimal body temperature and body surface temperature was measured using an infrared laser thermometer and adjusted to match awake mice. The tumors were measured manually using parallel segmentation in the Vevo2100 software (v1.6.0, VisualSonics Inc.). Table 3 shows the typical settings used to capture the tumors using 3D ultrasound.

#### 2.4.3. Ultrasound Treatment

**Figure 2:**
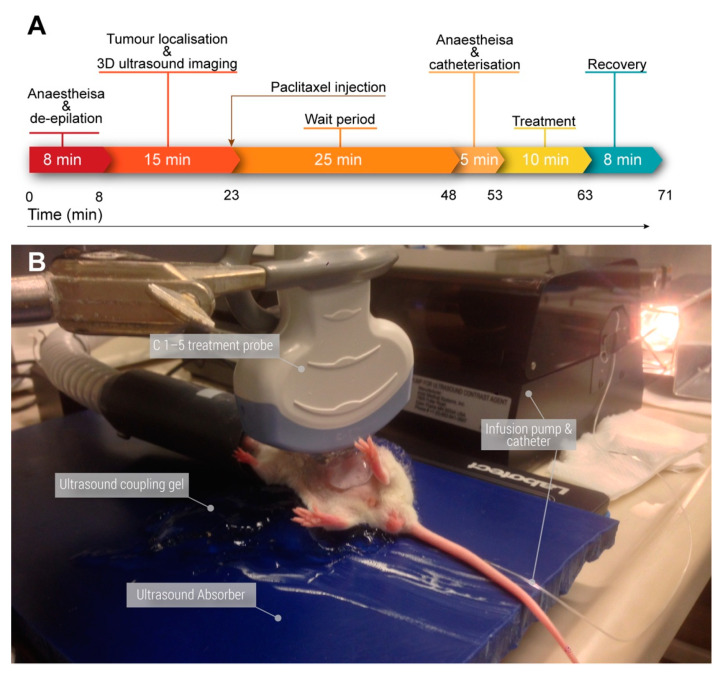
Timeline and setup for treating a mouse using the diagnostic ultrasound probe. (**A**) A typical timeline where the entire imaging, treatment, and recovery procedure lasted 71 min. (**B**) The treatment configuration where bubbles are infused via the tail vein and ultrasound is applied via the C1-5 probe.

The timing of a single imaging and treatment cycle is shown in Figure 2. Mice were split into 6 groups shown in Table 4 based on initial bioluminescence counts via the greedy algorithm for the partition problem resulting in no significant difference between the groups when evaluated via a one-way ANOVA.

Following the 3Rs of ethical research and current EU directives [51], microbubble-only groups were not included, as this was not expected to induce any additional bioeffects [52]. Microbubble and ultrasound alone groups were not included due to experimental limitations in treating and imaging more mice in the same period.

Following ultrasound imaging, mice were injected IP with 8 mg/kg Paclitaxel (Hospira, Norway). After 20–25 min, mice were re-anesthetized using isoflurane in air and catheterized with a 30 G needle connected to a 22 cm long microbore tubing (Saint-Gobain™ Tygon™ ND 100-80) using an illuminated tail vein restrainer. Patency was verified using saline. While still anesthetized, the mice were moved to an ultrasound coupling gel covered, heated, ultrasound-absorbing pad (Aptflex F28, Precision Acoustics) with a contact surface temperature of 37 °C. The catheter was subsequently connected to a microbubble filled 1 mL syringe (BD Plastipak Ref: 303172, BD Bioscience) in an oscillating microbubble perfusion pump (VueJect^®^, Bracco Spa, Milan, Italy). Using this 1 mL syringe resulted in a flow multiplier of 0.058×, meaning to achieve the required flow rate of 20 µL/min a flow rate of 0.35 mL/min was needed. This was rounded to 0.4 mL/min due to the minimum increment step size, i.e., 23 µL/min. The microbubbles were diluted in 50 mg/mL glucose water (B. Braun, Melsungen, Germany) to match concentrations and increase stability. 

Ultrasound coupling gel heated to 39 °C was placed on the abdomen of the mice and the C1–5 probe was lowered perpendicular to the spine (c.f., Figure 2). The probe was positioned to avoid mouse compression. This resulted in a typical imaged tumor depth of 0.5–1.5 cm. Additional ultrasound gel was used between the probe and mouse if the tumor depth was less than 1 cm. The kidney, liver, and spine were used as markers to identify the location of the tumor based on the prior visualization using the Vevo2100. 

The infusion pump was started and after 45 s of infusion the 10 min treatment countdown timer was started. A delay of 45 s was introduced to clear the saline dead space. This resulted in a total injected IV volume of 250 µL per mouse. Following treatment completion mice were kept under a heat lamp until awakened.

During ultrasound imaging and treatment, the mouse body surface temperature was measured using a non-contact IR temperature gauge (Clas Ohlson, Oslo, Norway). A body surface temperature of 30–32 °C was sustained as this matched the surface body temperature of ambulatory mice.

#### 2.4.4. Ultrasound Treatment Settings

Ultrasound emission was characterized using a 200 µm diameter PVDF needle hydrophone (Precision Acoustics Ltd.) connected to 200 MHz digitizing oscilloscope (WaveJet 324a, Lecroy) in a custom made 3-axis 220-L calibration tank using degassed water at 22 °C. The ultrasound transmission surface was coated with a thin bead of ultrasound transmission gel (ECO Supergel, Ceracarta, Forli, Italy) and subsequently encased in a probe cover (Pasante Healthcare Ltd., West Sussex, UK). The ultrasound transmission gel bead was subsequently compressed and spread to cover the entire transmission surface resulting in a thin film of gel and the entire probe was immersed in the water tank. The transducer was optically aligned with the hydrophone axis and several 1-D scans were performed to optimize the alignment. Field characterization was performed by moving the hydrophone along the x-, y-, and z-axes and saving the detected waveforms and parameters.

The acoustic output parameters were calculated using an attenuation of 0.3 dB/MHz/cm in MATLAB (MathWorks, Natick, MA, USA). 

The E9 in combination with the C1-5 probe was set into the combined Doppler and contrast mode as per guidelines by GE. The settings shown in Supplemental Table S1 were used. Supplemental Table S2 describes the on-screen parameters depicted at these given settings. Table 5 summarizes the acoustic output for the chosen ultrasound settings. Examples of the waveforms, calculated acoustic intensity, and frequency content of each pulse used to image and treat the mice are shown in Supplemental Figure S5. Supplemental Figure S6 shows the 2D field scans of both the imaging and treatment pulses.

#### 2.4.5. Tumor Measurements

Tumor volume and percentage vascularization was quantified using the VEVO software (v1.6.0, Visualsonics Inc.). The tumor volume was determined using manual segmentation and the parallel slice method. Three users with experience in abdominal ultrasound imaging were blinded to the groups and segmented the tumors. The users then compared the tumor selection and if the selection did not match the three users discussed until a mutual agreement was met. Tumor volumes and corresponding vascularization percentages of a single user were used in all subsequent data plotting and analysis. Tumor percentage vascularization was determined from the power Doppler signal and is defined as the percentage of segmented tumor volume that contained a power Doppler signal.

### 2.5. Statistical Analysis

Results are expressed as mean values ± SEM. In the in vitro studies, comparisons were made using a two-way ANOVA where the variables were either ultrasound intensity and microbubble concentration, or microbubble type and concentration. Multiple comparisons were made using Turkeys multiple comparisons across ultrasound intensity and microbubble type, respectively. In the preclinical studies, comparisons between groups were made using a two-tailed unpaired Student t-test or one-way ANOVA with Dunnett’s multiple comparisons. Bioluminescence was evaluated using the area under each curve and compared using a one-way ANOVA with a Kruskal–Wallis test as the data was not normally distributed. Tumor growth inhibition was calculated using the slope method [53]. Differences with *p* < 0.05 were considered as statistically significant. Statistics were analyzed using GraphPad PRISM v8.3.0 (GraphPad Software Inc., La Jolla, CA, USA) software. 

## 3. Results

### 3.1. Microbubble Characterization

#### 3.1.1. Size, Concentration and in Vial Stability

**Figure 3:**
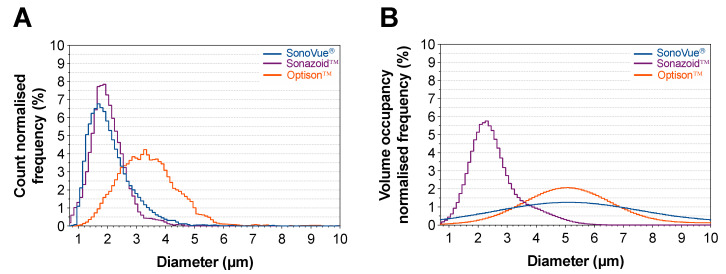
Size distribution histograms of SonoVue^®^, Sonazoid™, and Optison™ measured optically. (**A**) The histogram normalized to the number of microbubbles counted. (**B**) Normalizes each bin to the total volume occupied by all the microbubble population. The cumulative sum of each histogram is 100%.

To define optimal conditions for sonoporation, first microbubbles were thoroughly characterized. Size distribution of the three microbubble agents was assessed using optical microscopy immediately after reconstituting or opening the vial (Figure 3). The results are presented with a bin size of 0.1 µm. A general overview of the measured properties can be seen in Table 6. When evaluating the size distribution using count normalized frequency (Figure 3A) SonoVue^®^ and Sonazoid™ have a similar size distribution with the peak size (median diameter) of 1.7 and 1.9 µm, respectively. Optison™ was the larger microbubble agent with a peak size (median diameter) of 3.3 µm. Based on this characterization, Sonazoid™ was the most monodispersed particle with a d_90_/d_10_ of 2.06, followed by Optison™ at 2.15, and SonoVue^®^ at 2.46.

Alternatively, volume normalization of the data presents a significantly different picture (Figure 3B). Using this metric, Sonazoid™ has the smallest peak diameter at 2.3 µm whilst SonoVue^®^ and Optison™ were near double the size with a peak diameter of 5.1 µm. The polydispersity order remained the same but the polydispersity of Sonazoid™ was less than half compared to SonoVue^®^ and Optison™.

**Figure 4:**
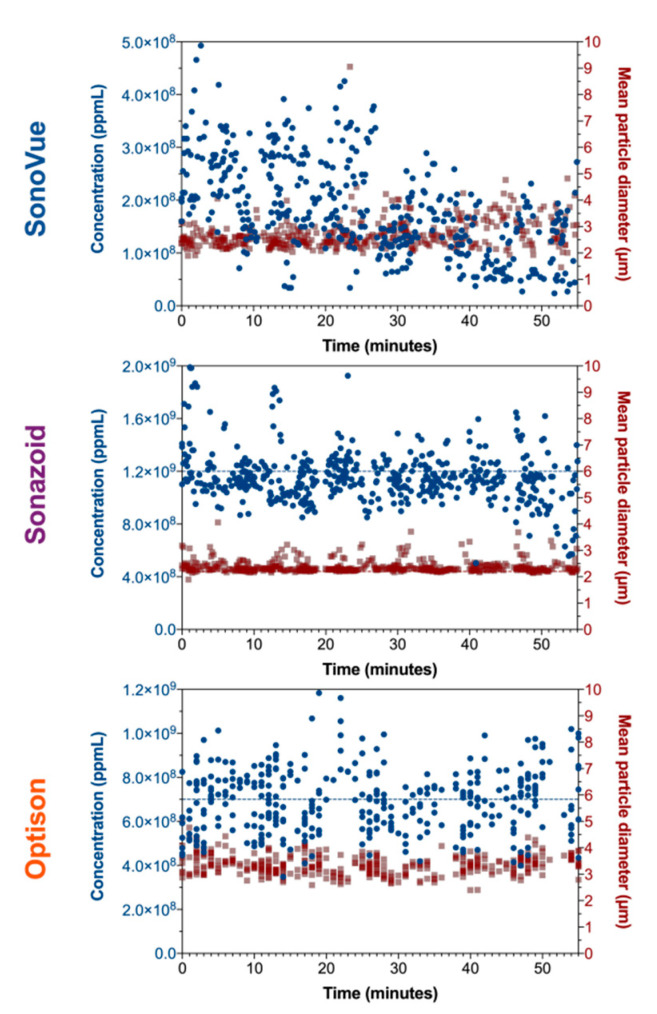
Concentration and mean size as a function of time for SonoVue^®^, Sonazoid™, and Optison™ measured optically. SonoVue^®^ had the largest loss in microbubbles after 30 min where the median concentration was halved and the largest change in microbubble size and variance. Both Sonazoid™ and Optison™ showed minimal loss in concentration and change in size.

Of the three agents Sonazoid™ was the most concentrated with a median concentration of 1.27 × 10^9^ ppmL when fresh, followed by Optison™ at half the concentration (0.61 × 10^9^ ppmL) then SonoVue^®^ (0.24 × 10^9^ ppmL) being five times less concentrated than Sonazoid™. After keeping the vials at room temperature for 30 min, the concentration of SonoVue^®^ was halved (*p* < 0.0001). Sonazoid™ showed a decrease in concentration of 12% (*p* < 0.0001) whilst no difference was observed for Optison™ (Figure 4 and Figure S7).

All microbubble agents showed a decrease in roundness after 30 min, with Optison™ showing the largest change (from 0.92 to 0.88) but none of the changes were significant (Supplemental Figures S8 and S9). The mean diameter of the measured populations also changed after 30 min in both SonoVue^®^ and Optison™. In SonoVue^®^ the mean diameter increased from 2.51 to 3.20 µm (*p* < 0.0001) and decreased in Optison™ from 3.55 to 3.02 µm (*p* < 0.0001) (Table 6).

#### 3.1.2. Attenuation and Cavitation Measurements

**Figure 5:**
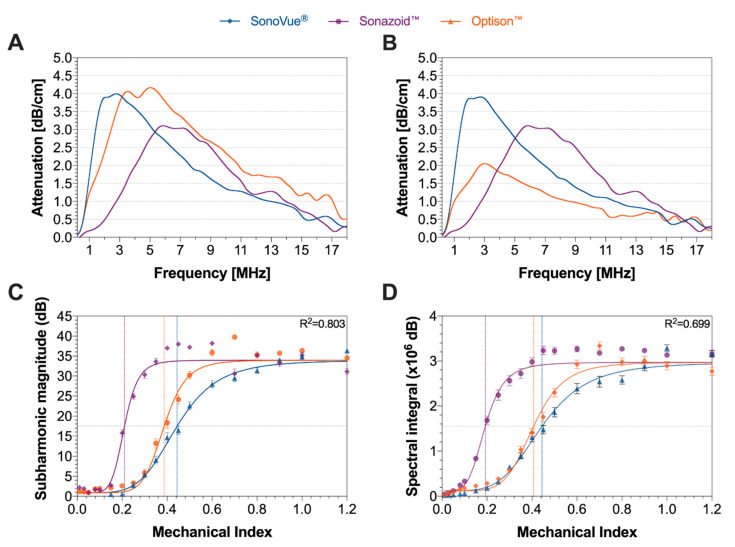
Acoustic measurements of the three ultrasound contrast agents. (**A**) The attenuation as a function of frequency for all agents at an identical particle concentration of 75 × 10^3^ ppmL. (**B**) Shows the same data, but the concentration has been adjusted that all agents have the same cross-sectional area. (**C**) Shows the subharmonic magnitude between 400–600 kHz, emitted from the microbubbles when excited by a 1 MHz, 25 cycle burst pulse, while (**D**) shows the same results but evaluating the spectral integral.

Further characterization was performed, analyzing the attenuation of the three ultrasound contrast agents. Figure 5A presents the results at a concentration of 75 × 10^3^ ppmL. This concentration represents 1.88 mL of SonoVue^®^, 0.31 mL Sonazoid™, or 0.54 mL Optison™ in the 5L blood pool of humans. 

The bulk resonance frequency was based on the frequency at which the peak attenuation is observed. At the given concentration SonoVue^®^ had the lowest resonance frequency of 2.78 MHz at 4.00 dB/cm, followed by Optison™ (5.10 MHz at 4.17 dB/cm) and lastly Sonazoid™ (5.89 MHz at 3.11 dB/cm). As acoustic attenuation is strongly correlated to both microbubble elasticity and cross-sectional area, the concentrations were normalized so that all agents have the same cross-sectional area using the highest concentrated agent (Sonazoid™ as the baseline) (Figure 5B). Following this transformation, the matched concentrations were 62 × 10^3^ ppmL for SonoVue^®^ and 24 × 10^3^ ppmL for Optison™. At these concentrations, we see that the bulk resonance frequency of Optison™ drops to 3.02 MHz and the attenuation decreased to 2.05 dB/MHz. The frequency response of SonoVue^®^ remained similar with a minor drop in attenuation as expected due the lower concentration. Normalizing the cross-sectional area indicates that SonoVue^®^ is the most elastic agent as it has the highest attenuation, whilst Optison™ is the most ridged of the three, with the lowest attenuation; approximately half of SonoVue^®^.

The sub-harmonic magnitude and spectral integral of the side-scattered ultrasound for a given MI for all three ultrasound contrast agents are shown in Figure 5C,D. The horizontal grey dashed line shows the point of inflection for all the agents. The vertical lines that intercept the grey line are the suggested cavitation threshold MIs for each of the agent. The exact MIs at the point of inflection (i.e., the cavitation threshold) for each of the agents are summarized in Table 7. For both, the subharmonic magnitude and spectral integral, a similar trend can be observed where Sonazoid™ had the lowest cavitation threshold at an MI of 0.19 or 0.21 followed by Optison™ at 0.39 or 0.41 and SonoVue^®^ at 0.44. At MIs above 1.2 a decrease in subharmonic magnitude and spectral integral was observed due to the microbubbles being destroyed in the nearfield of the HIFU transducer and attenuating the ultrasound from reaching the acoustic focus of the measuring PCD.

Based on the sigmoidal curve, the cavitation behavior can be split into three regions: stable, transitional, and inertial (Supplemental Figure S10).

### 3.2. Simulations

#### 3.2.1. Microbubble Pharmacokinetics

When an injected microbubble concentration of 2.4 × 10^8^ ppmL was used, simulations indicated that in the pre-clinical experiments the blood bubble concentration would range between 1.5 and 6.2 × 10^6^ ppmL during the ultrasound treatment depending on the bubble half-life (Supplemental Figure S11). Therefore, in the in vitro studies, concentrations between 0.29 and 6.72 × 10^6^ ppmL were used to cover this range. We simulated using a microbubble concentration of 2.4 × 10^8^ ppmL because this is the measured mean concentration of the SonoVue^®^, which is the least concentrated ultrasound contrast agent.

When using microbubbles with a half-life of 30s the infusion reached a steady state after 4.5 min. Microbubbles with a half-life greater than 90s did not reach steady state even after 10 min. 

#### 3.2.2. Simulated Microbubble Behavior

An animation of the simulated response of the three bubbles tested is shown in Supplemental Videos 2 and 3. In these videos, Panel A shows the ultrasound pulse and the moving cursor shows the point in time that is being simulated. The black circle in Panel B shows the response of a single bubble at mean diameter of the given microbubble agent whilst Panel C shows the response of a single bubble at the median diameter of the given microbubble agent. In each frame in Panels B and C the green circles show the minimum bubble diameter, and the red circles show the maximum bubble diameter until the current time point. For ease of visualization, horizontal dotted and dashed lines have been placed at top and bottom of these circles. In Supplemental Video S2, the bubbles are reacting the to a typical B-mode ultrasound pulse. It can easily be seen at the end of the video that all three bubbles respond differently to the same ultrasound pulse. As expected, the softest bubble, SonoVue^®^, has the largest volumetric oscillations. It can also be observed that the 2.5 µm SonoVue^®^ bubble has a large phase shift in relation to the ultrasound pulse as the ultrasound pulse is above its resonance frequency, i.e., the bubble cannot respond fast enough to the ultrasound so when the acoustic pressure is positive the bubble is still expanding, and the opposite when the acoustic pressure is negative. This is not observed to the same extent for Sonazoid™ or Optison™.

In Supplemental Video 3, the same bubbles are reacting to the treatment pulse. This treatment pulse has a lower frequency and more ultrasound cycles, but the acoustic amplitude is similar to the B-mode pulse. Once again, SonoVue^®^ shows the largest oscillation amplitude and Optison™ the smallest. In general, all bubbles oscillate in phase to the ultrasound pulse as it is below their resonant frequency. In addition, the SonoVue^®^ and Sonazoid™ bubbles are seen to transiently pause at their equilibrium radius between ultrasound cycles resulting in highly nonlinear oscillations.

The results of the microbubble simulations are depicted in Supplemental Figure S12. The data is presented as a function microbubble diameter. Panels A and B (Supplemental Figure S12) show the oscillation amplitude, i.e., the difference between the maximum and minimum diameter, as a function of bubble equilibrium diameter for each microbubble type and ultrasound pulse. When excited by the B-mode pulse, there is only a small difference between SonoVue^®^ and Sonazoid™. Specifically, in SonoVue^®^ the maximum oscillation amplitude is observed at a bubble diameter of 3.4 µm with an amplitude of 0.718 µm whilst for Sonazoid™ this was observed at a bubble diameter of 3.7 µm with an amplitude of 0.752 µm. In contrast, Optison™ had a maximum oscillation amplitude when the bubble diameter was 6.8 µm with a maximum amplitude of 0.593 µm.

When excited by the treatment pulse, a similar but amplified response was seen. In this case, due to the lower ultrasound frequency the maximum oscillation amplitude was observed in the larger bubbles which were closer to resonance. For all bubble the oscillation amplitude was much larger than with the B-mode pulse. The B-mode pulse resulted in maximum oscillation amplitudes of ~22% of the equilibrium diameter for SonoVue^®^ and Sonazoid™ and 9% for Optison™. The treatment pulse resulted in maximum oscillation amplitudes of 38%, 42% and 28% of the equilibrium diameter for SonoVue^®^, Sonazoid™ and Optison™, respectively.

In Supplemental Figure S12C–F the same data is shown, but for each bubble diameter the response has been multiplied by the frequency or volume occupancy normalized size distribution (i.e., Figure 3A,B) to normalize for the number of bubbles available at a given size. The results indicate that the acoustic pulse was able to induce large microbubble oscillations in the primary population of SonoVue^®^ and Sonazoid™ whilst less of a response was observed in Optison™. In the panels in Columns 2 and 3, the overall efficacy of each agent based on the given metric is estimated to be the area under the curve.

**Figure 6:**
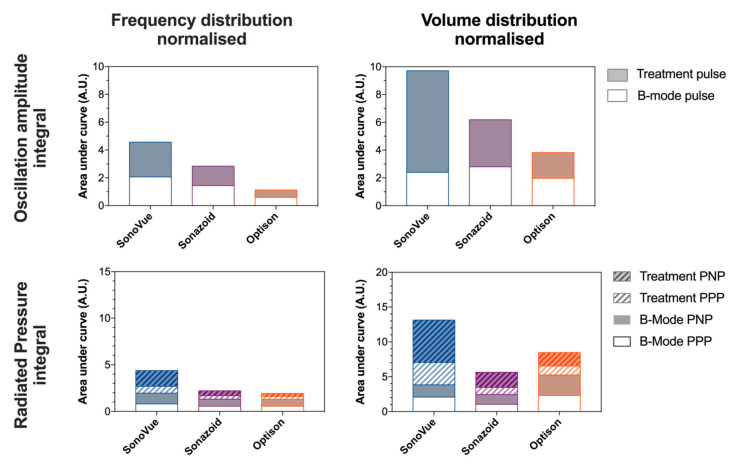
Summary of simulation results. For the used ultrasound conditions, SonoVue^®^ induced the largest cumulative oscillation amplitude and radiated pressure, i.e., potentially having the largest effect on the surrounding cells. The treatment pulse resulted in the largest cumulative oscillation amplitude and radiated pressure for SonoVue^®^ and Sonazoid™ but not for Optison™ indicating that the treatment pulse was suboptimal for the size disruption of Optison™. PNP: Peak-Negative Pressure; PPP: Peak-Positive Pressure.

The peak-negative pressure of a microbubble for given size at a distance of 7 µm from the center of the bubble, i.e., the assumed distance of a capillary wall, are shown in Supplemental Figure S12G,H. The equivalent peak-positive pressure same is shown in Supplemental Figure S12M,N. Following frequency distribution normalization (Supplemental Figure S12I–L,O,P) SonoVue^®^ has the highest peak and the largest area under the curve followed by Sonazoid™, then Optison™. In contrast when normalizing using the volume occupancy distribution (Supplemental Figure S12K,L,Q,R) Optison™ has the highest peaks during the B-mode pulse, but once again SonoVue^®^ had the highest peak during the treatment pulse. A summary of the simulations as integrated function of the diameter is presented as a stacked bar chart (Figure 6). When evaluating the oscillation amplitude as a metric for sonoporation efficacy, the simulations indicate that independent of normalization method (frequency or volume occupancy) SonoVue^®^ would be the most effective agent, followed by Sonazoid™ then Optison™. A similar trend is observed when using the radiated acoustic pressure as a metric; SonoVue^®^ would be the most effective agent, but depending on the normalizing method, either Sonazoid™ or Optison™ could be the second- or third-best agent.

### 3.3. In Vitro Experiments

Next, we tested the three different microbubble agents in vitro. Three different pancreatic cell lines were treated and the efficacy of sonoporation was assessed by uptake of a cell impermeable dye, calcein. Supplemental Figure S13 shows an example of the flow cytometry results for the MIA PaCa-2 cells treated with Sonazoid™ at a concentration of 2.8 × 10^6^ ppmL. A small increase in calcein-positive cells was observed in the MIA PaCa-2 and PANC-1 cell lines for all three microbubbles even if no ultrasound was applied, but in the BxPC-3 cell line this was only observed for SonoVue^®^. This indicates that the microbubbles themselves induces uptake of calcein in the respective cell lines.

**Figure 7:**
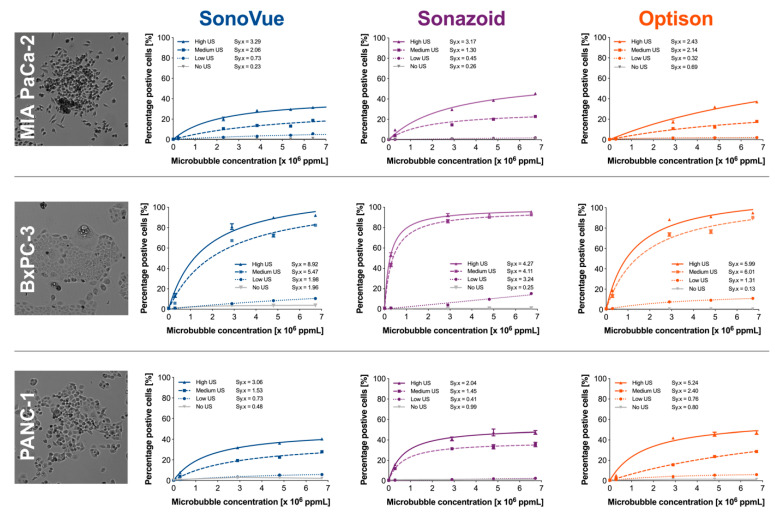
Results of in vitro experiments evaluating the percentage of cells affected by sonoporation using SonoVue^®^, Sonazoid™, and Optison™ at different acoustic conditions, different microbubble concentrations, and three difference pancreatic cancer cell lines. In general, increasing the acoustic power and microbubble concentration increased the number of calcein-positive cells. All three cell lines responded differently with BxPC-3 being the most sensitive, followed by PANC-1 and MIA PaCa-2 being the least sensitive.

All three cell lines (MIA PaCa-2, BxPC-3, and PANC-1) showed different responses when exposed to a specific ultrasound and microbubble condition (Figure 7). Overall, the responses to sonoporation followed the expected “dose–response” behavior where there was a rapid increase in calcein-uptake followed by an eventual plateau at the larger microbubble concentrations. In general, BxPC-3 was the most sensitive cell line reaching the highest calcein-positive population with all microbubble formulations. The highest percentage of calcein-positive cells was achieved when using Sonazoid™; 96% of the BxPC-3 cells were calcein-positive (92% and 95% for SonoVue^®^ and Optison™, respectively), in contrast to 45% for MIA PaCa-2 (32% and 37% for SonoVue^®^ and Optison™, respectively), and 47% for PANC-1 cells (40% and 47% for SonoVue^®^ and Optison™, respectively).

Within each cell line as well as each microbubble agent, both increasing the ultrasound intensity (i.e., ultrasound pressure and pulse length) and microbubble concentration resulted in a significantly (*p* < 0.0001) higher proportion of calcein-positive cells. This was observed for all tested cell lines. In addition, the interaction between concentration and ultrasound intensity was significant in all cases (*p* < 0.0001).

When comparing within each cell line and ultrasound power (no US, low, medium, and high) both concentration and type of microbubbles in all conditions was significantly different (*p* < 0.0001)—except for BxPC-3 when exposed to low ultrasound power (*p* = 0.084). The interaction between ultrasound intensity and microbubble concentration was also significant across all comparisons (*p* < 0.0001). In all conditions and across all cell lines there was always a single agent that was significantly better.

Ultrasound alone resulted in no additional calcein uptake vs. control independent of the ultrasound settings used (Supplementary Figure S13, top row). This was consistent across all three cell lines. This indicates that the increased delivery of calcein was due to the combination of ultrasound and microbubbles and not either parameter by itself.

**Figure 8:**
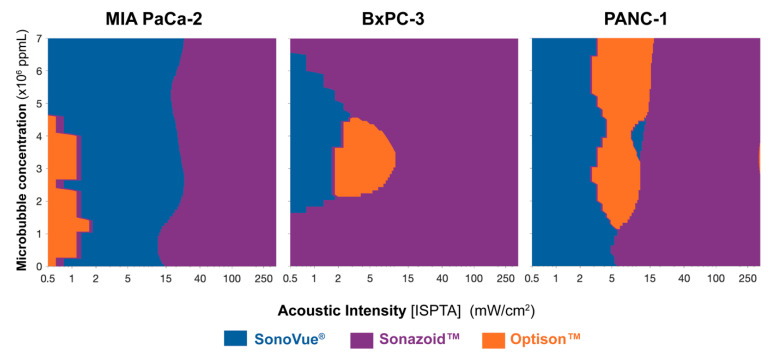
Compiled results showing which microbubble is the best at a given ultrasound intensity and microbubble concentration for the three cell lines MIA PaCa-2, BxPC-3, and PANC-1. The color at a given point indicates which microbubble had the largest amount of calcein-positive cells. Each cell line has a different sensitivity pattern to the microbubble type and acoustic intensity. SonoVue^®^ or Optison™ are typically best at lower intensities (<15 mW/cm^2^) but Sonazoid™ was best at higher ultrasound intensities (>15 mW/cm^2^).

The data on which bubble is best for a given ultrasound power and microbubble concentration across each cell line is summarized in Figure 8. The intermediary values were calculated using a cubic interpolation in MATLAB. For each value on the grid, the best bubble is colored according to the legend, i.e., blue for SonoVue^®^, purple for Sonazoid™, and orange for Optison™.

The general trend observed across all three cell lines is that SonoVue^®^ is the best microbubble below 15 mW/cm^2^ (I_SPTA_). Sonazoid™ is the best at acoustic intensities above 15 mW/cm^2^ (I_SPTA_). At certain conditions, (below 15 mW/cm^2^ (I_SPTA_)), Optison™ appears to be a better agent. Nevertheless, the multiple comparisons indicate that the difference between Optison™ and SonoVue^®^ was not statistically significant. Therefore, Figure 8 could also be represented with SonoVue^®^ and Sonazoid™ only, whereby SonoVue^®^ is replacing the Optison™ region.

### 3.4. In Vivo Experiments

#### 3.4.1. Body Weight

**Figure 9:**
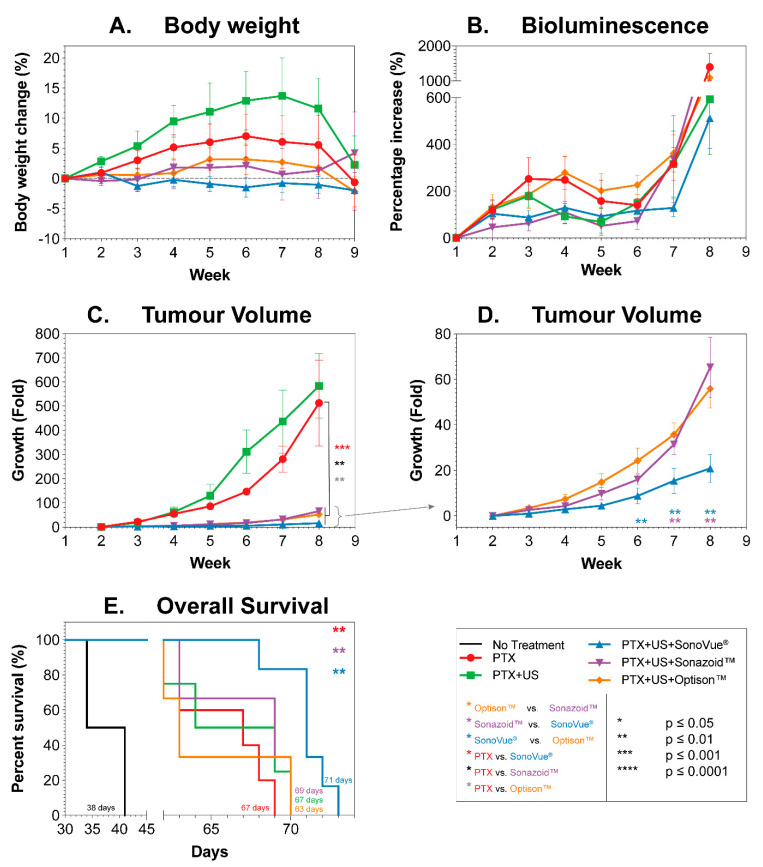
Quantitative metric of treatment and therapeutic response as a function of time. (**A**) The total bodyweight for all mice as a function of time. No mice showed a drop below the 20% body weight loss limit indicating the treatment did not have significant adverse effects. (**B**) The percentage increase in whole body bioluminescence as a function of time. Bioluminescence images were taken 2–3 days before treatment. An initial increase in bioluminescence is observed followed by a decreased due to necrotic cores forming. (**C**) The tumor volumes measured with 3D ultrasound. All microbubble groups showed significant growth inhibition. (**D**) The three groups treated with microbubbles to help better distinguish between groups. SonoVue^®^ showed significant tumor inhibition vs. Optison™ and Sonazoid™. (**E**) The overall survival of all groups. The number at the bottom of the groups indicates the median survival. The mice treated with SonoVue^®^ showed the longest survival. This survival was significant vs. drug alone and both other bubbles.

When comparing different microbubble agents in vivo, body weight was used as a proxy for systemic toxicity and adverse effects. Figure 9A shows the mean body weight change (%) of the different groups as a function of time, normalized to the pre-treatment body weight. A body weight loss of > 20% indicates severely increased toxicity or stress.

Treatment with the different regimes did not result in any large weight loss (i.e., no loss <5%) when compared to the starting body weight in any of the animals. When comparing the overall mean body weight to paclitaxel (PTX) treatment alone, adding SonoVue^®^+US resulted in a significant decrease (*p* = 0.008) while adding ultrasound alone to the paclitaxel treatment resulted in a significant mean increase in body weight (*p* = 0.018) (One-Way ANOVA).

Based on the low percentage in body weight loss, the results indicate that the treatment does not add additional stress to the mice and can be considered safe.

#### 3.4.2. Bioluminescence

Bioluminescence data, as measure of tumor load, are shown in Figure 9B. The bioluminescence has been normalized using the pre-treatment value as the baseline. In all groups, a general development profile of a slow increase followed by a dip leading onto a rapid increase was observed. There was no significant difference in bioluminescent counts throughout the study at individual time points. Using the area under the curve as a proxy for overall tumor burden showed that only SonoVue^®^ was significantly better than PTX (*p* = 0.0397) and Optison™ (*p* = 0.0123).

**Figure 10:**
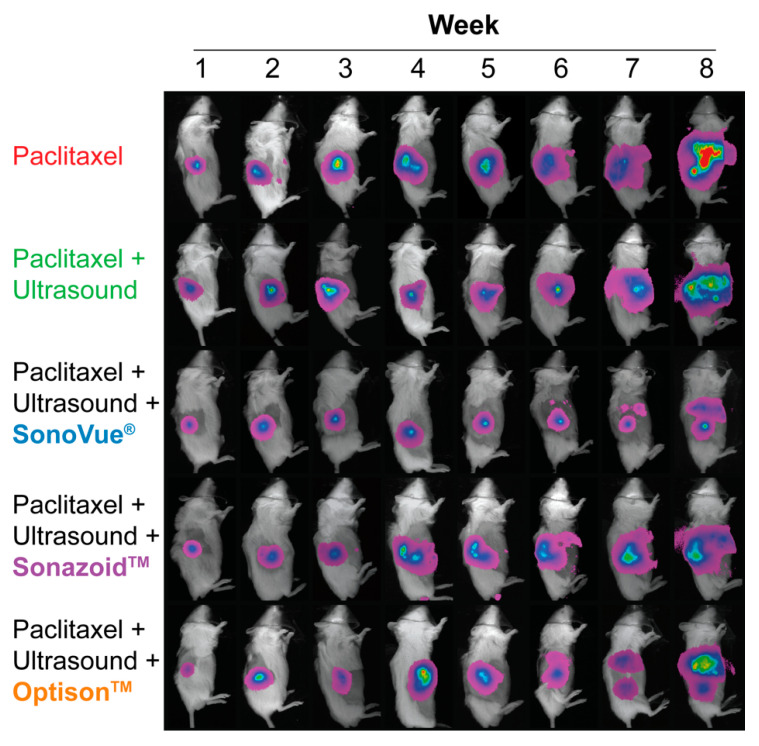
Qualitative photographs of bioluminescence as a function of time. Each row is a single mouse in which the bioluminescence count matched the group mean closest at week 5. At the end of treatment, the bioluminescence spread engulfs the entire abdomen of the mice treated with paclitaxel alone or paclitaxel with ultrasound. In contrast, the mice treated with sonoporation seem to have a more localized cancer spread (i.e., primary tumor and liver), potentially due to the slower growth.

Figure 10 shows a qualitative overview of the bioluminescence spread throughout the course of treatment. Over the first two weeks of treatment (week 2 and 3) the tumor can be visualized as a single point/lesion. In week 3 (after 2 treatments) in some of the mice two distinct spots can be observed, indicating tumor spread into adjacent organs or tissue. As time progressed the bioluminescent area increases in all mice. At the end of treatment (week 8), the mice treated with PTX showed substantial abdominal spread of the bioluminescence indicating metastatic spread throughout the abdominal and chest cavity. A very similar spread was observed for the mice treated with PTX + US or in combination with Sonazoid™ or Optison™. In contrast the mice treated with PTX + US + SonoVue^®^ show the least amount of bioluminescence spread. Correlating the location of the bioluminescence to the internal organs indicates metastatic spread primarily in the liver. 

#### 3.4.3. Tumor Volume

Tumor volumes were measured using 3D ultrasound. Normalized tumor volumes as a function of time are depicted in Figure 9C,D. Figure 9C shows the mean volumes for all groups, while Figure 9D highlights the groups treated with microbubbles. The two control groups (PTX and PTX+US) showed the fastest growth. The group treated with PTX+US indicates faster tumor growth but this is due to a single outlier with rapid growth (detected via Grubb’s test at week 3 onwards). There was no significant difference between these groups at any time point, whether including or excluding the outlier. All groups treated with microbubbles had a significantly inhibited tumor growth vs. PTX alone; *p* = 0.0008, 0.0036, and 0.0015 for SonoVue^®^, Sonazoid™, and Optison™, respectively (one-way ANOVA). When comparing between microbubble types, Figure 9D, there was no significant difference between Sonazoid™ and Optison™ at any point during treatment. SonoVue^®^ was significantly better than Sonazoid™ (*p* = 0.006–0.0082) and Optison™ (*p* = 0.0041–0.0096) from week 6 onwards (i.e., after 3 treatments).

**Figure 11:**
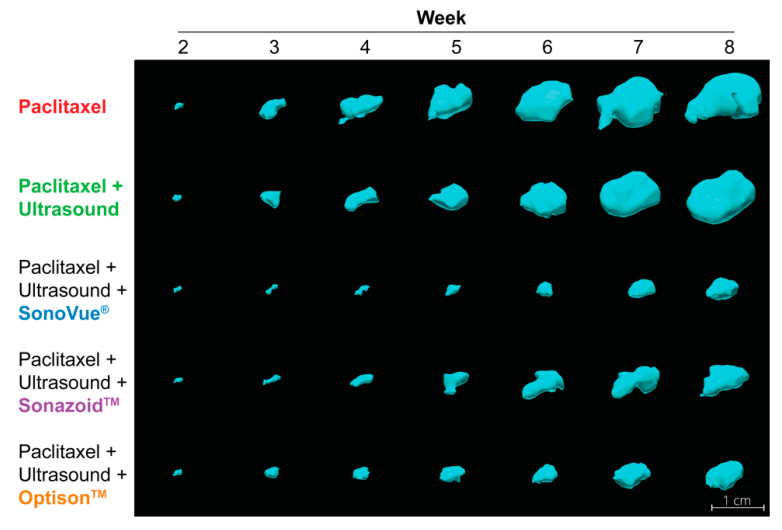
Qualitative renderings of the tumor volumes measured using 3D ultrasound. Each row is a single mouse in which the tumor volume matched the group mean closest at week 8. All tumors have a similar size before treatment (week 2) and the difference in tumor volume development is clearly observable over the following 7 weeks.

Figure 11 shows 3D rendering of tumors from each group that best matched the mean tumor volumes at week 8. The difference in tumor volumes can be observed from week 3 (after a single treatment) where the groups treated with microbubbles are visibly smaller than the groups with PTX alone or PTX+US. This difference is clearly observable up to the end of treatment. Supplemental Figure S14 shows the tumor volumes of the remaining mice at week 8 with the tumor growth inhibition indicated above each group. All groups showed tumor growth inhibition when using this method emphasizing that all three microbubbles were effective at enhancing the therapeutic efficacy of PTX.

#### 3.4.4. Vascularization

**Figure 12:**
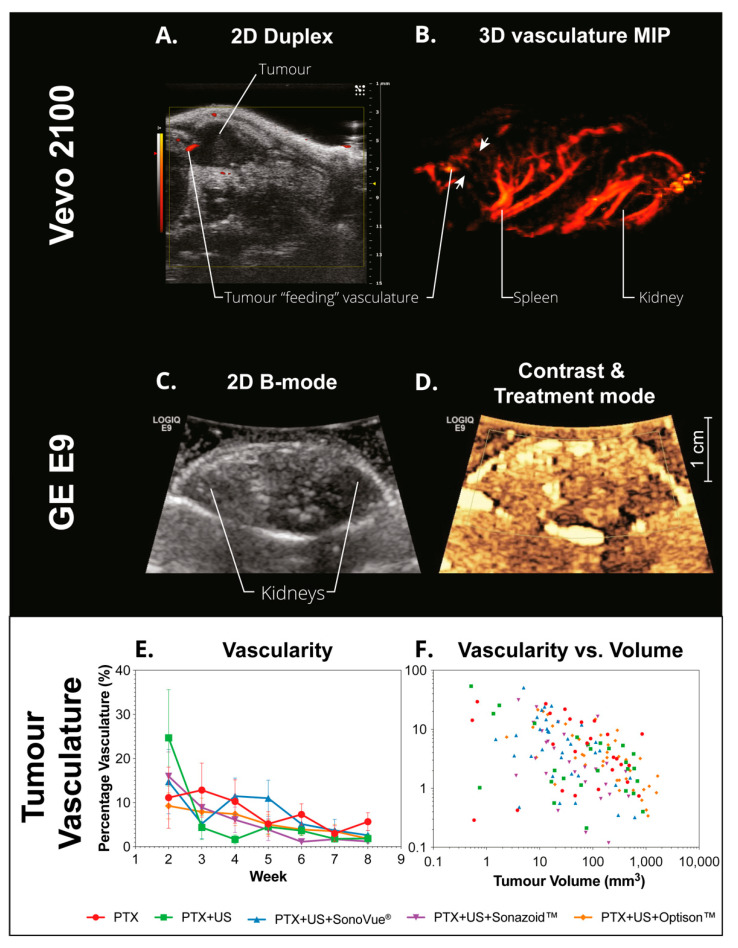
Example ultrasound images and quantitative results from tumor vascularization measurements. (**A**) An example image of the PDAC tumor imaged with duplex mode (B-mode and power doppler). The tumor can clearly be delineated, and part of the feeding vasculature is detected. (**B**) A Maximum Intensity Projection (MIP) of the power doppler signal following 3D imaging. The tumor is located between the two white arrows. The feeding vessel is clearly visible in addition to microvasculature throughout the tumor. Other organs such as the spleen and kidney can also be clearly distinguished. (**C**) A B-Mode ultrasound image (3 MHz) of a healthy mouse with no tumor or contrast agent captured using the GE E9 scanner and C1-5 ultrasound probe to help depict the image quality and resolution. The landmarks used to locate the pancreas and tumor (such as the kidneys) can be easily delineated. (**D**) The same mouse but during treatment. A significant loss in image quality is observed due to the different imaging mode. (**E**) The quantitative results of the tumor percentage vasculature (calculated from the doppler content of the 3D tumors) as a function of time. No difference was observed between the groups. (**F**) The same data with every datapoint plotted. A general trend of decreasing vascular as a function of tumor volume is observed but no distinct correlations are observed.

Independent of the metric and correlation used, there was no significant difference or correlation between the groups treated with sonoporation vs. US or PTX alone. Figure 12 shows an example of the 3D Doppler images captured with the Vevo 2100 (Figure 12A,B). The primary feeding vessel can be seen on both images. This feeding vasculature was observed in all tumors. Figure 12B shows a maximum intensity projection of the abdominal scan where the vasculature of the tumor, spleen, and kidney can be seen. The amount of vasculature in the tumor is substantially less than that of the spleen and kidney; in addition, it is less than the surrounding pancreatic tissue. Figure 12E,F shows the percentage of vasculature as a function of time or tumor volume, respectively. The general trend observed was that the percentage of tumor vasculature decreased with increasing tumor volume, indicating the tumors were growing faster than the vasculature.

#### 3.4.5. Survival 

The overall survival of all groups is shown in Figure 9E. The use of PTX without sonoporation increased median survival from 38 days (no treatment) to 67 days (PTX). Despite the significant reduction in tumor volume when adding Sonazoid™ or Optison™ to the treatment, this did not result in significantly longer survival than PTX alone or PTX+US. The mice treated with SonoVue^®^+US+PTX showed the longest survival (71 days), which was significantly longer than vs. PTX (*p* = 0.0036, 67 days), PTX+US (*p* = 0.0101, 67 days) and both Sonazoid™+US+PTX (*p* = 0.0095, 69 days) and Optison™+US+PTX (*p* = 0.0044, 63 days). 

## 4. Discussion

### 4.1. Microbubble Characterization

Overall, the microbubble characterization procedure showed a good match with the data available in literature and provided from the manufacturers (Supplemental Table S3). Based on this characterization, Sonazoid™ could be considered the best agent for diagnostic imaging as it has the narrowest size distribution, the longest in-use stability, and produced a nonlinear signal during low-frequency excitation at lower acoustic amplitudes when compared to SonoVue^®^ and Optison™. Nevertheless, an ideal imaging agent does not necessarily fit the requirements for an optimal sonoporation agent.

Choosing an appropriate method to determine the size distribution is quite critical as the bulk resonance frequency and attenuation will be better depicted by the volume normalized size distribution. Comparing Figure 3B and Figure 5B, a strong correlation between peak size and attenuation can be observed, where Optison™ and SonoVue^®^ have a similar resonance frequency as they have a similar normalized size distribution, and Sonazoid™ has a higher resonance frequency due to its smaller size. Nonetheless, in the linear regime, the most favorable condition for the transfer of energy from an ultrasonic field to a microbubble occurs when the center frequency of the ultrasonic field equals the resonance frequency of the bubble [54]. Therefore, while the volume normalization allows for a better understanding of microbubble-induced attenuation and the exitance of the larger microbubbles, it is not expected that the typically small number of larger microbubbles in most formulations will have the predominant impact on sonoporation. The count normalization allows for better optimization of ultrasound parameters to largest population of microbubbles, potentially resulting in better therapeutic effect.

The lack of (or variability) in the stability of SonoVue^®^ and Optison™ is an important parameter that will have a large impact on in vitro and in vivo experiments. As indicated by the in vitro experiments, the microbubble concentration has a significant impact on sonoporation efficacy. In addition, as gasses escape the microbubbles, or microbubbles change shape and size this will also affect how they respond to ultrasound, e.g., in a more non-linear fashion, or the shell may become stiffer resulting in lower amplitude oscillations. 

The decrease in roundness of Optison™ can be linked to the stiffer shell. Specifically, as Optison™ is composed of polypeptides, it is more ridged as all the peptide bonds possess a partial double bond character. Therefore, as the microbubble loses gas content to the surrounding fluid the shell may only deform at weaker joints resulting in a non-spherical and buckled microbubble. In contrast SonoVue^®^, composed of frequently available flexible ether linkages, the PEG chains impart greater flexibility. This allows the entire microbubble shell to deform at multiple points as gas is lost, maintaining a more spherical shape. As the microbubbles become less spherical this may impact the volumetric oscillation amplitude, the resonance frequency, and the cavitation threshold potentially resulting in reduced therapeutic efficacy.

Based on our measurements, we suggest that neither SonoVue^®^ nor Optison™ should be used 20–30 min after re-constituting, opening, or removing from the fridge. In particular, in the case of SonoVue^®^, this is a shorter in-use time than recommended by the manufacturer (Supplemental Table S3).

In current ultrasound and microbubble mediated therapy, especially at higher-acoustic powers, the spectral content is used to determine if the microbubbles are undergoing inertial cavitation [55,56]. 

Our cavitation threshold measurements indicate that such spectral content can be observed even at an MI of 0.2 to 0.6. While we hypothesized that the softer microbubbles (SonoVue^®^) would undergo inertial cavitation (based on the spectral profile) at a lower MI than the harder microbubbles (Sonazoid™ and Optison™) this was not observed [57]. In fact, Sonazoid™ reached the cavitation threshold at approximately half the MI when compared to SonoVue^®^ and Optison™, for both metrics evaluated. Based on the simulation work presented, we assumed that the spectral content appears due to the microbubble oscillations becoming nonlinear at lower acoustic pressures, rather than the induction of inertial cavitation. In addition, Sonazoid™ had the steepest slope in the MI vs. spectral content graphs (Figure 5C,D) followed by Optison™ and SonoVue^®^. The slope correlated to the volume normalized polydispersity (Table 5) indicating that as the MI increased bubbles of specific sizes surpass the non-linear oscillation threshold.

In our work here, these measurements were made to gain a better understanding of the microbubble behavior during the in vitro and in vivo work. Based on this data we assume that in the in vitro experiments, the low ultrasound setting is in the stable regime for all agents, the medium ultrasound is in the stable inertial regime for Sonazoid™ only [44], and all agents are in the inertial cavitation regime for the high ultrasound intensity.

While these data are indicative, blood gas saturation, protein content, temperature, and pulsatile flow affect stability and half-life of the microbubbles, thus these measurements should not be directly translated into an in vitro or in vivo situation. 

The cavitation measurements were performed at a different center frequency and pulse repetition frequency compared to the in vitro and in vivo experiments. Different frequencies are expected to change the behavior, stability, and cavitation threshold of the microbubbles [58,59], thus further work is needed to confirm the regime the bubbles were in for each specific scenario.

### 4.2. Simulations

#### 4.2.1. Microbubble Pharmacokinetics

The microbubble pharmacokinetic simulations indicated that the in vivo stability (i.e., half-life) of the microbubbles has a large impact on the potential concentration, meaning that even if identical concentrations are injected IV, bubbles with longer half-lives will result in significantly higher concentrations. Assuming partial correlation with the in vitro experiments, where a higher microbubble concentration showed enhanced model drug uptake, this indicates that a microbubble with a longer in vivo stability should be more effective for sonoporation. Nevertheless, the in vivo results showed that this was not the case or a dominant factor at the given treatment conditions. Further work should be performed to verify the in vivo microbubble concentrations, stability, and size distribution.

#### 4.2.2. Simulated Microbubble Behavior

Simulating microbubble behavior indicated that (for the metrics evaluated) SonoVue^®^ would be the best bubble as it induces the highest stresses on cells when compared to Sonazoid™ and Optison™. This matched the results from the in vitro and in vivo studies. The in vitro studies indicated that at low acoustic intensities (<15 mW/cm^2^) SonoVue^®^ would be the optimal ultrasound contrast agent. Looking up this point and correlating the injected concentrations in Figure 8 (MIA PaCa-2 panel) shows that indeed SonoVue^®^ was the most effective agent. A similar result was observed in the in vivo studies where SonoVue^®^ inhibited tumor growth most significantly and resulted in the longest overall survival.

Whilst these correlations seem very promising, the simulations assumed that the bubbles were in a static environment with no blood flow with no nearby boundaries, such as vessel walls, or any nearby interacting microbubbles. The model used assumes the microbubbles are perfectly spherical and does not include thermal effects. This means that for non-spherical microbubbles, as seen for Optison™, the simulation may not be representative as the volumetric oscillations would not be symmetrical, potentially resulting in lower induced cell stress, especially at low acoustic pressures. The thermal effects, specifically the transfer of heat from the shell to gas within the microbubbles is also expected to further dampen the volumetric oscillations [60] indicating that at the higher acoustic pressures the stressing induced may be overestimated. 

At higher microbubble concentrations, the microbubbles interact, creating clusters and coalescing. This can result in a change in shell thickness, bubble size, and bulk resonance frequency, significantly modifying their response to a given ultrasound field.

Furthermore, the simulations do not take into account inertial cavitation, i.e., if a microbubble cavitates it may generate a shockwave inducing more pressure in the surrounding tissue, release its encapsulated gas, or not induce any further interaction will the cells to enhance therapeutic efficacy. 

Therefore, these simulations can only be considered a simple start for computational models of understanding sonoporation efficacy.

### 4.3. In Vitro Experiments

The in vitro experiments performed in this study indicate that both the type of microbubble and microbubble concentration, the ultrasound intensity, as well as the cell type all affect sonoporation efficacy. Considering the results from the simulations, it can also be assumed that other acoustic conditions (e.g., center frequency, pulse bandwidth, and pulse repetition frequency) will affect the sonoporation efficacy. Unfortunately, due to the design of the plate sonicator, it was not possible to change the center frequency or pulse bandwidth to evaluate these parameters. It can be assumed that better matching the resonance frequency to Sonazoid™, would improve its effectivity at lower concentrations. Additionally, broadening the pulse bandwidth, matching the polydispersity of SonoVue^®^ or Optison™, would result in a better performance of these agents at higher acoustic amplitudes.

Previously, we showed in vivo that using ultrasound alone at similar acoustic settings resulted in significant increase in drug uptake in mouse kidneys (healthy tissue) [61]. This was not observed here and may be due to the difference in cell line and response to stresses, or the lack of correlation between in vitro experiments and healthy tissue in vivo.

Whilst the in vitro experiments showed strong reproducibility, they do not represent a replacement for in vivo studies. However, they are a good method to refine and validate theoretical expectations of sonoporation efficacy.

The primary experimental limitation in this setup is that a cell impermeable dye, such as calcein, does not match the pharmacokinetics and distribution behavior of actual drugs [48]. In addition, in this work the microbubbles are in direct contact with the cells, a behavior that may not be true in vivo, unless the blood flow is slow, and/or the acoustic radiation force is sufficient. Our experimental setup also used hard plastic surface to grow cells. This interface is not a total acoustic reflector generating standing waves but it reflected 10–20% of the ultrasound and thereby potentially caused nodes of higher acoustic pressures or unpredictable acoustic fields. Furthermore, there was no pulsatile flow or temperature control.

### 4.4. In Vivo Experiments

Sonoporation with the addition of any of the microbubble formulations in combination with PTX significantly reduced tumor growth, whilst only SonoVue^®^ was able to significantly enhance the efficacy of PTX on overall survival of the mice. At the ultrasound intensity (16 mW/cm^2^) used in the preclinical study SonoVue^®^ perfomed best, as hypothesized from the simulations and in vitro study. As the ultrasound parameters must match the physiochemical properties of the microbubbles used, this may be a possible explanation of the additional improvement of PTX using SonoVue^®^ + US in this study vs. gemcitabine + SonoVue^®^ + US in a previous study (I_SPTA_ = 688 mW/cm^2^) using the same orthotopic model [16]. This may also be due to the differences in the pharmacokinetics of the drugs, i.e., paclitaxel vs. gemcitabine.

Only the group treated with SonoVue^®^+US showed a significant weight loss during treatment. A potential reason behind this may be due to imperfect tumor targeting and enhanced therapeutic efficacy of PTX. Specifically, when placing the ultrasound on the mice, other organs such as intestines, spleen, and stomach, may be subjected to collateral ultrasound, hence the systemic effect manifesting as weight loss. This problem would be less of an issue in the clinical scenario where the patients are much larger, and the tumors can be precisely targeted.

While all the tumor volumes showed a significant benefit in adding sonoporation to the treatment regime, the bioluminescence data showed no significant response at specific time points. The initial increase in bioluminescence is correlated to the initial tumor formation and rapid cell division whilst the tumor still had substantial blood supply. Following this tumor establishment period, a necrotic core is formed, and the tumor progressively becomes hypoxic resulting in less adenosine triphosphate (ATP) production and producing a false “drop” in bioluminescence. The subsequent rapid increase can be correlated to metastatic spread as whole body bioluminescence was measured and the tumor cells adapting to the environment. Therefore, this metric could have potential for indicating when a tumor becomes necrotic but using bioluminescence alone would not allow for differentiation between necrotic tumor or tumors that have reduced in volume.

Whilst all sonoporation treated mice showed inhibited tumor growth only SonoVue^®^ resulted in increased survival. Based on the bioluminescence measurements (Figure 9B and Figure 10) this may be due to metastatic spread seen in the other groups.

Very few tumors showed a spherical shape when measured using 3D ultrasound. This obscure shape is due to the limited space available for the tumors to grow. In addition, as the tumors grew, they fused onto surrounding organs and tissue, and within each tissue the growth rate varied. Eventually multiple metastatic sites and the primary tumor also fuse resulting in a continuously evolving primary tumor. Nonetheless, when using bioluminescence as a proxy for overall tumor burden, SonoVue^®^ was significantly better than the drug alone; matching tumor volume and survival data.

As sonoporation is predominantly a biomechanical effect on the tumor vasculature, it was expected to have an impact on the amount of active vasculature. This was not observed in this study potentially due to the method used to evaluate the vasculature. Power Doppler, whilst very sensitive, is limited by the blood flow rate, meaning it cannot visualize very slow-moving blood in the smallest capillaries. The doppler settings also need to be optimized for each mouse to compensate for artifacts from breathing and other abdominal movement. In addition, imaging was performed one week after treatment and the vascular effects may be transient. Nevertheless, the results here show that this treatment did not have a long-lasting effect on the large vasculature, such as long-term vascular damage. While a more optimal way to evaluate the capillaries would be to use contrast-enhanced ultrasound, this was not possible in our study due as additional anesthesia would induce more stress on the animals and the additional microbubble and ultrasound imaging may be a confounding factor.

The vascular imaging did indicate that the tumors grew in a similar manner to that seen in the clinical counterpart, i.e., rapid growth in which the vasculature is unable to keep up, resulting in tumors that exhibit low vascularization at the late stage of detection. 

A major difficulty in this experiment was ensuring the primary tumor was treated, in addition to how the tumors were delineated. For tumor boundary detection, three blinded users delineated the tumor. The delineations were then compared, and any major discrepancies were evaluated till mutual agreement. As it was difficult to locate the tumors at the earliest stages, tumor delineation was started at the last week of treatment and performed backwards to the first image. This method allows for the use of landmarks for identifying the tumor.

As the tumor could only be clearly visualized on the clinical ultrasound scanner once it was larger than 0.5 cm in diameter at the initial stages of treatment, the abdominal scar from the surgical procedure was used to align the ultrasound probe along with the location of the kidney and spleen (Figure 12C,D).

Whilst all attempts were made to ensure correct treatment and tumor delineation there is always a potential for error. 

A major limitation of this study is the small number of animals. Due the heterogeneous nature of the orthotopic tumor subsequent studies should use more animals per group or more homogenous and predictive models.

## 5. Conclusions

A strong correlation between simulations, in vitro, and in vivo studies was observed for determining the optimal ultrasound contrast agent for specific ultrasound conditions. In vitro studies indicate that at low acoustic intensities SonoVue^®^ was the best agent, but at higher intensities Sonazoid™ was best agent and resulted in the highest number of cells with model drug uptake. Simulations were able to predict that for the defined ultrasound conditions SonoVue^®^ would be the best ultrasound contrast agent closely followed by Sonazoid™ and Optison™.

Preclinical studies indicate that sonoporation can be performed using any commercial ultrasound contrast agent and diagnostic ultrasound imaging system to significantly enhance the therapeutic efficacy of paclitaxel for treating PDAC in an orthotopic murine model. At the specific ultrasound conditions, SonoVue^®^ was the optimal ultrasound contrast agent.

## Figures and Tables

**Table 1:** Physicochemical properties of microbubbles and blood used to perform simulations.

Property	Unit	Abbreviation	SonoVue^®^	Sonazoid™	Optison™
Shell stiffness	N/m	χ	0.22 [34,35,36]	0.53 [37]	4.00 [38,39]
Specific heat ratio of gas [40]	A.U.	γ	(SF_6_) 1.0934	(C_4_F_10_) 1.0699	(C_3_F_8_) 1.0731
Gas compressibility [41]	×10^−6^ m^2^/N	κ	9.173	6.537	7.458
Surface tension	N/m	σ	0.052 [35,42]	0.042 [43,44]	0.9 [45]
**Mouse Blood**					
Fluid density	kg m^3^	ρ	1 057 [46]		
Liquid viscosity	mPa s	ν	5.996 [47]		

**Table 2:** Ultrasound conditions used to treat the cells using the plate sonicator.

Name	Frequency (MHz)	No. of Cycles	Duty Cycle (%)	Pulse Repetition Frequency (Hz)	MI	Intensity	
						I_SPTA_ (mW/cm^2^)	I_SPPA_ (W/cm^2^)
Low	2.00	20	0.4	22	0.10	3	1
Medium	2.00	80	1.8	22	0.20	50	3
High	2.00	160	3.6	22	0.39	358	10

**Table 3:** Typical Power Doppler settings for measuring active tumor vasculature.

Parameter	Value
Frequency (MHz)	32
Power (%)	100
PRF (kHz)	2
Gate	1
Doppler Gain (dB)	35
2D Gain (dB)	22
Depth (mm)	Variable
Width (mm)	14.08
Beam Angle (º)	0
Sensitivity	5
Line Density	Full
Persistence	Off
ECG/Resp Gate	Off/On
3D range	Variable
3D step	Variable

**Table 4:** Summary of groups used in the in vivo study. All microbubble groups included 6 mice due to the expected variance. Two mice with no treatment were included to verify the efficacy of the paclitaxel treatment. Drug used was Paclitaxel at 8 mg/kg injected IP.

Group	Microbubble	Dilution	Ultrasound	Drug	*n*
1	-		- –	-	2
2	-		- -	✓	5
3	-		✓		4
4	SonoVue^®^	None	✓		6
5	Sonazoid™	1:4	✓		6
6	Optison™	1:1	✓		6

**Table 5:** Acoustic settings used to enhance drug delivery. Values have been attenuated by 0.3 dB/MHz/cm.

Centre Frequency (MHz)	Duty Cycle (%)	Mechanical Index	Acoustic Intensity, I_SPTA_ (mW/cm^2^)	Peak Negative Acoustic Pressure (MPa)
1.8	1.1%	0.2	16	0.268

**Table 6:** Summary of measured microbubbles parameters using optical microscopy. Errors are SEM.

Microbubble	Diameter (µm), Percentage (%), PSD d_90_/d_10_		Fresh➝After 30 min		
	Count Normalized	Volume Normalized	Concentration (×10^9^ ppmL)	Roundness	Mean Diameter
SonoVue^®^	1.7 µm, 6.8%, 2.46	5.1 µm, 1.3%, 6.36	0.239 ± 0.015→ 0.126±0.013	0.94→0.93	2.51 ± 0.07→ 3.20 ± 0.18
Sonazoid™	1.9 µm, 7.8%, 2.06	2.3 µm, 5.8%, 2.50	1.269 ± 0.035→ 1.111 ± 0.039	0.95→0.94	2.45 ± 0.08→ 2.43 ± 0.07
Optison™	3.3 µm, 4.2%, 2.15	5.1 µm, 2.1%, 4.89	0.607 ± 0.061→ 0.609 ± 0.118	0.92→0.88	3.55 ± 0.19→ 3.02 ± 0.07

**Table 7:** Cavitation threshold MI for the three microbubble formulations determined by the subharmonic magnitude and spectral integral. The numbers in the brackets are the 95% confidence intervals. Both methods showed similar results.

Method	SonoVue^®^	Sonazoid™	Optison™
Subharmonic Magnitude	0.44 (0.440–0.453)	0.21(0.205–0.215)	0.39 (0.380–0.394)
Spectral integral	0.44 (0.433–0.456)	0.19 (0.185–0.200)	0.41(0.400–0.417)

## Data Availability

The data presented in this study are available on request from the corresponding author.

## Supplementary Materials

The following are available online at https://www.mdpi.com/article/10.3390/pharmaceutics14010098/s1, Figure S1: Schematic describing how the microbubble characteristics were measured using image analysis. Figure S2: Acoustic waveforms emitted by the PCD and HIFU transducer and their calculated frequency spectra with the peak transmit frequency labelled. Figure S3: Schematic illustration of how the microbubble radiated pressure was simulated. Figure S4: Gating strategy for determining the percentage of cells that were permeated with calcein. Figure S5: Acoustic pulses used to treat the animals. Figure S6: Field scans of the emitted ultrasound from the GE C1-5 ultrasound probe used during sonoporation treatment. Figure S7: Micrographs showing the change in concentration and size fresh and 30 minutes after reconstitution or opening the vials. Figure S8: Mean roundness of the three microbubble formulations as a function of time. Figure S9: Micrographs of the three microbubble formulations showing the shape of the bubbles when fresh, and after 30 minutes. Figure S10: Example frequency spectra obtained from each of the bubbles for the stable, transition, and inertial cavitation areas in the MI vs. Spectral parameter graphs. Figure S11: Simulated microbubble blood concentration as a function of time during continuous infusion. Figure S12: Simulation results showing the oscillation amplitude, peak-negative and -positive radiated pressure induced on a cell 7 µm away from the bubble centre induced from SonoVue®, Sonazoid™, and Optison™. Figure S13: Examples of flow cytometry measurements quantifying the percentage of MIA PaCa-2 cells that were calcein-positive. Figure S14: Violin plots show the size distribution of the mice remaining at week 8 with tumour growth inhibition based on median tumour volumes. Table S1: Parameters selected for inducing sonoporation. Table S2: On screen parameters using “optimised” sonoporation settings. Table S3: Characteristics of the commercial MBs used in the studies. Video S1: Rendering of design and construction of plate sonicator platform for performing in vitro sonoporation. Video S2: Simulated response of the three different microbubble formulation at key sizes to the B-mode ultrasound pulse. Video S3: Simulated response of the three different microbubble formulation at key sizes to the treatment ultrasound pulse.
