# Supplementary material for: SonoVue® vs. Sonazoid™ vs. Optison™: Which Bubble Is Best for Low-Intensity Sonoporation of Pancreatic Ductal Adenocarcinoma?
Source: Pharmaceutics. 2022 Jan 1;14(1):98. doi: 10.3390/pharmaceutics14010098 (PMC8777813; doi:10.3390/pharmaceutics14010098)
Supplement: Supplementary file 1 [file pharmaceutics-14-00098-s001.zip › pharmaceutics-1504598-supplementary/pharmaceutics-1504598-supplementary.pdf]

pharmaceutics

MDPI

Supplementary Materials: SonoVue® vs. Sonazoid™ vs. Optison™: Which Bubble Is Best for Low-Intensity Sonoporation of Pancreatic Ductal Adenocarcinoma?

Spiros Kotopoulis, Mihaela Popa, Mireia Mayoral Safont, Elisa Murvold, Ragnhild Haugse, Anika Langer, Georg Dimcevski, Christina Lam, Tormod Bjånes, Odd Helge Gilja and Emmet Mc Cormack

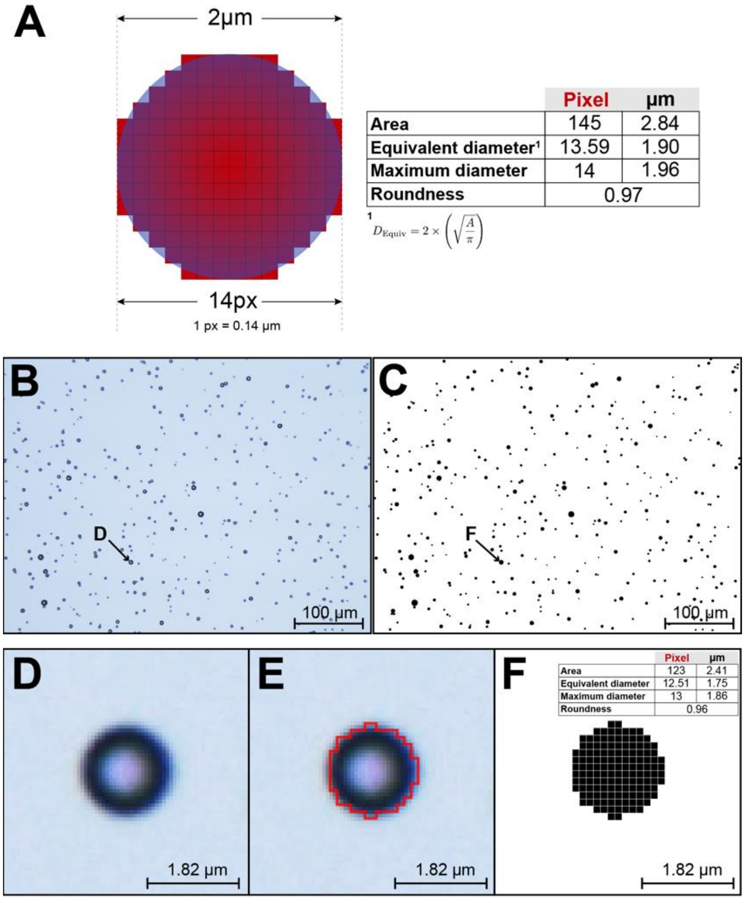

Figure S1. Schematic describing how the microbubble characteristics were measured using image analysis. **A** shows a graphic illustration of the most optimal detection of a 2-μm diameter bubble and the descriptive parameters. **B** shows a photomicrograph of Sonazoid™. **C** shows the microbubble detection results of the photomicrograph in (B), where each detected bubble is displayed as black pixels. **D** shows an image crop from B of a single microbubble. **E** overlays the detected microbubble outline (in red) over the photomicrograph to evaluate detection quality. **F** shows an image crop from C showing the single detected microbubble.

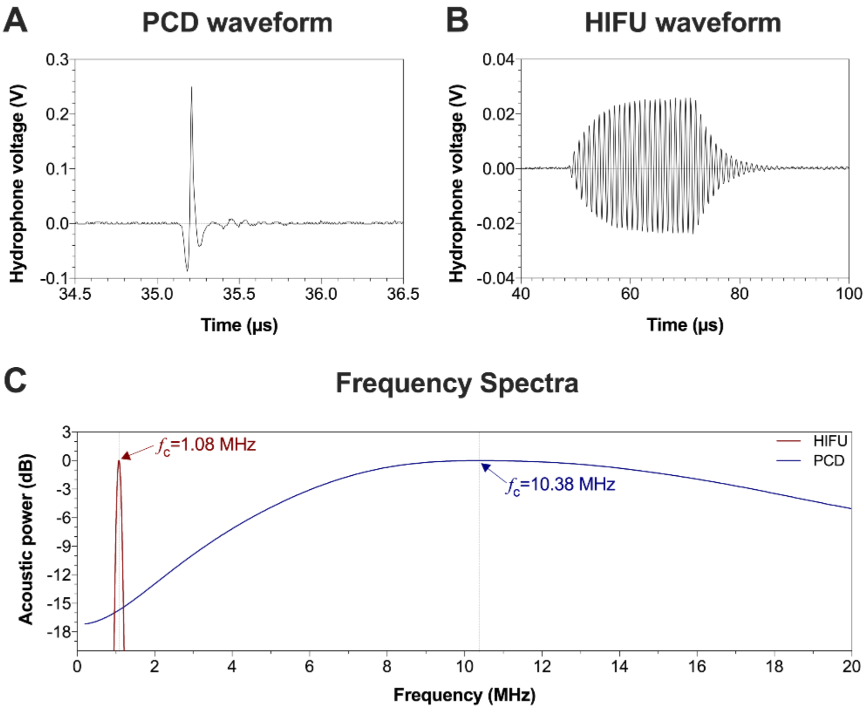

Figure S2. Acoustic waveforms emitted by the PCD and HIFU transducer (**A** and **B** respectively) and their calculated frequency spectra with the peak transmit frequency labelled (**C**).

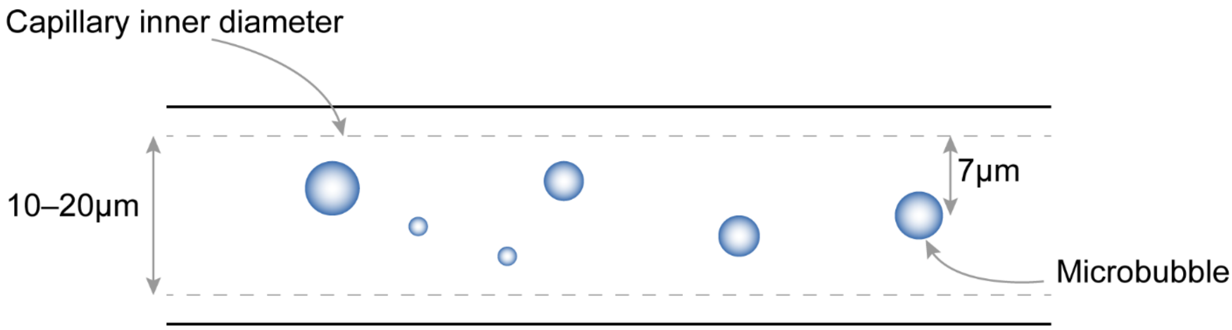

Figure S3. Schematic illustration of how the microbubble radiated pressure was simulated. The radiated acoustic pressure was simulated 7μm from the bubble centre mimicking a typical capillary in PDAC.

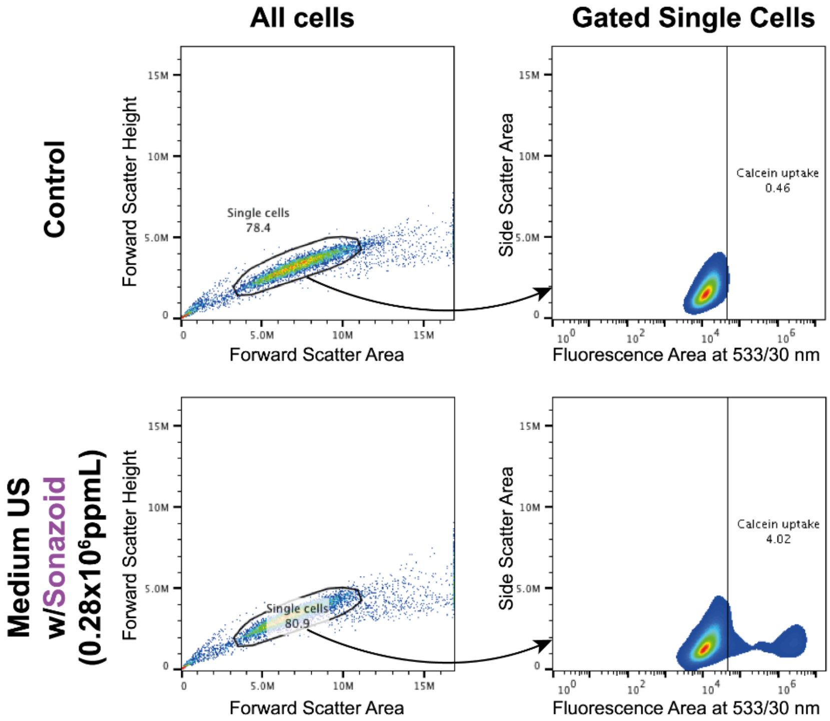

Figure S4. Gating strategy for determining the percentage of cells that were permeated with calcein.

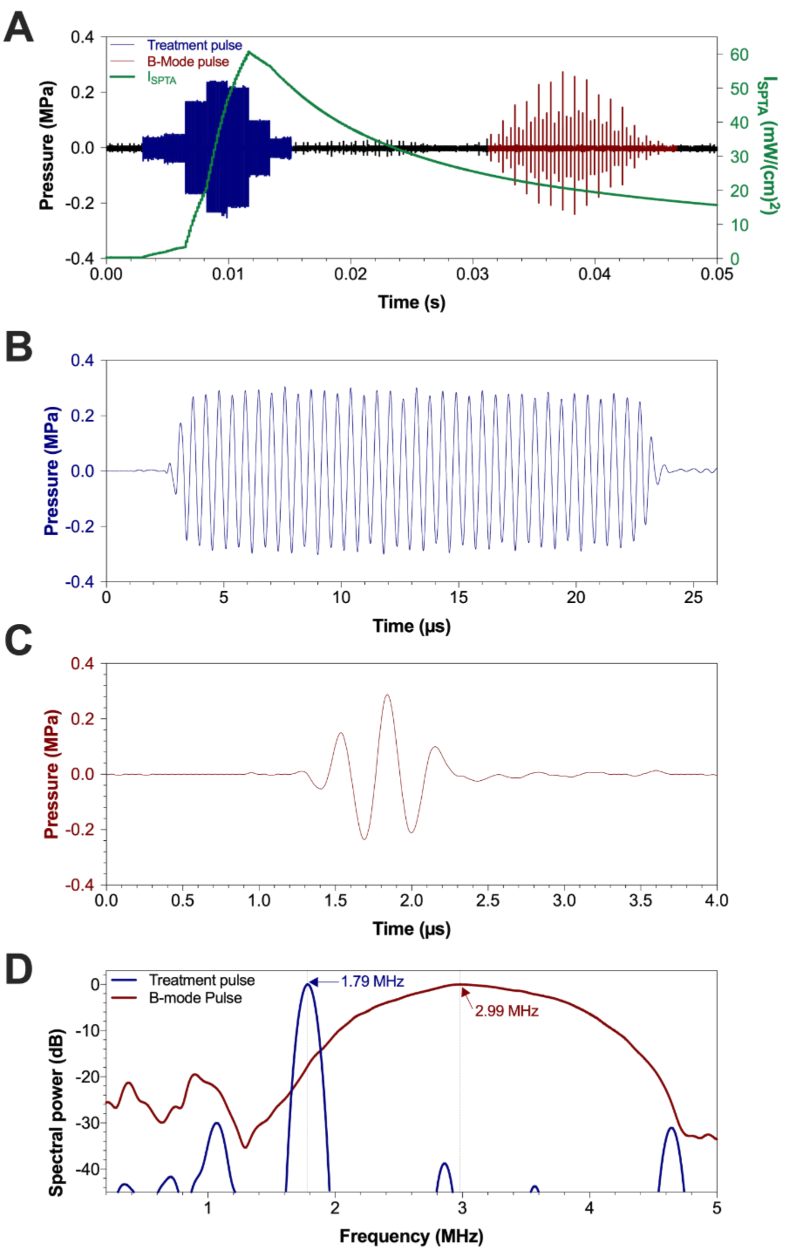

Figure S5. Acoustic pulses used to treat the animals. The blue pulse indicates the treatment pulse whilst the red pulse shows the B-mode imaging pulse. All measurements have been attenuated by 0.3 dB/MHz/cm.

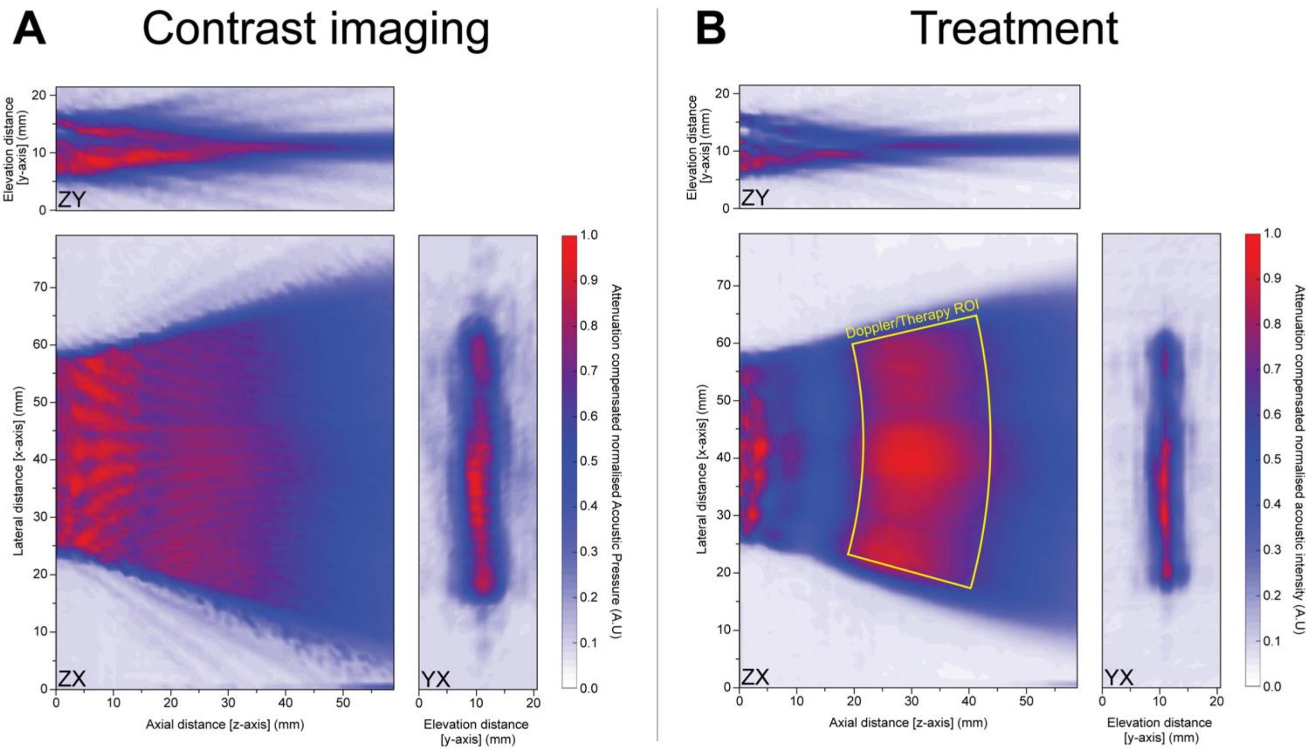

Figure S6. Field scans of the emitted ultrasound from the GE C1-5 ultrasound probe used during sonoporation treatment. **A** shows the beam pattern of the non-linear contrast imaging pulse (red, Supplemental Figure 5). **B** shows the beam pattern of the treatment pulse (blue, Supplemental Figure 5). The YX scans have been performed at the middle of the Doppler ROI (30 mm in the z-axis). All scans have been attenuated by 0.3 dB/MHz/cm.

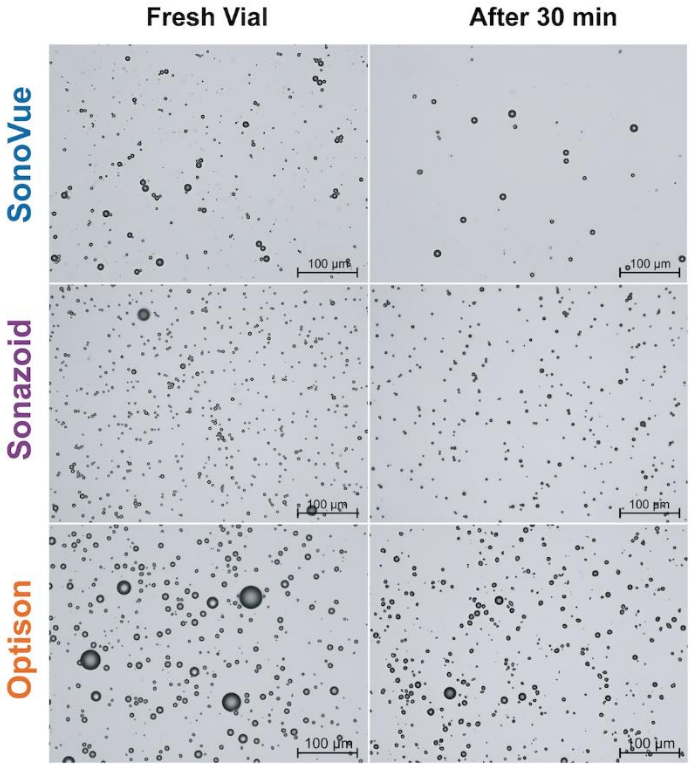

Figure S7. Micrographs showing the change in concentration and size fresh and 30 minutes after reconstitution or opening the vials. The largest change in concentration is seen in SonoVue®.

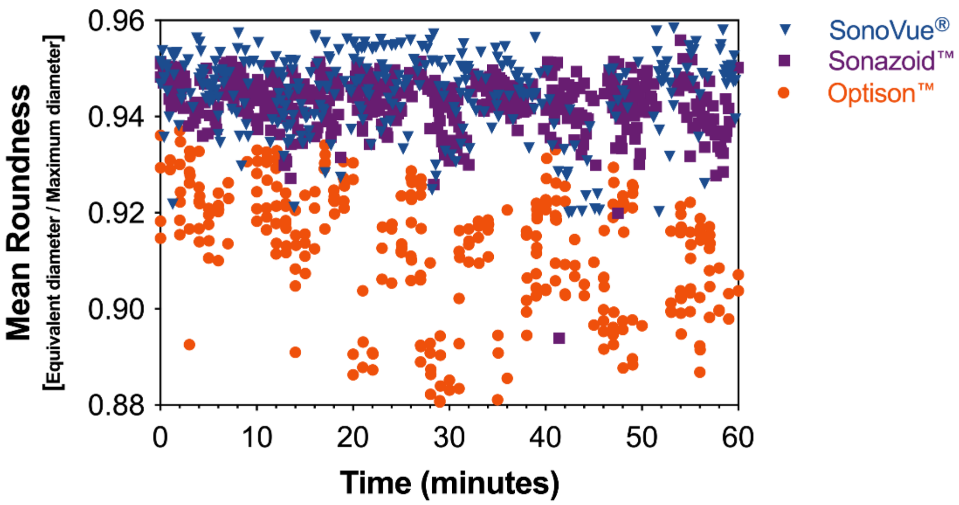

Figure S8. Mean roundness of the three microbubble formulations as a function of time. Optison™ exhibits a drop in mean roundness after removal from the fridge.

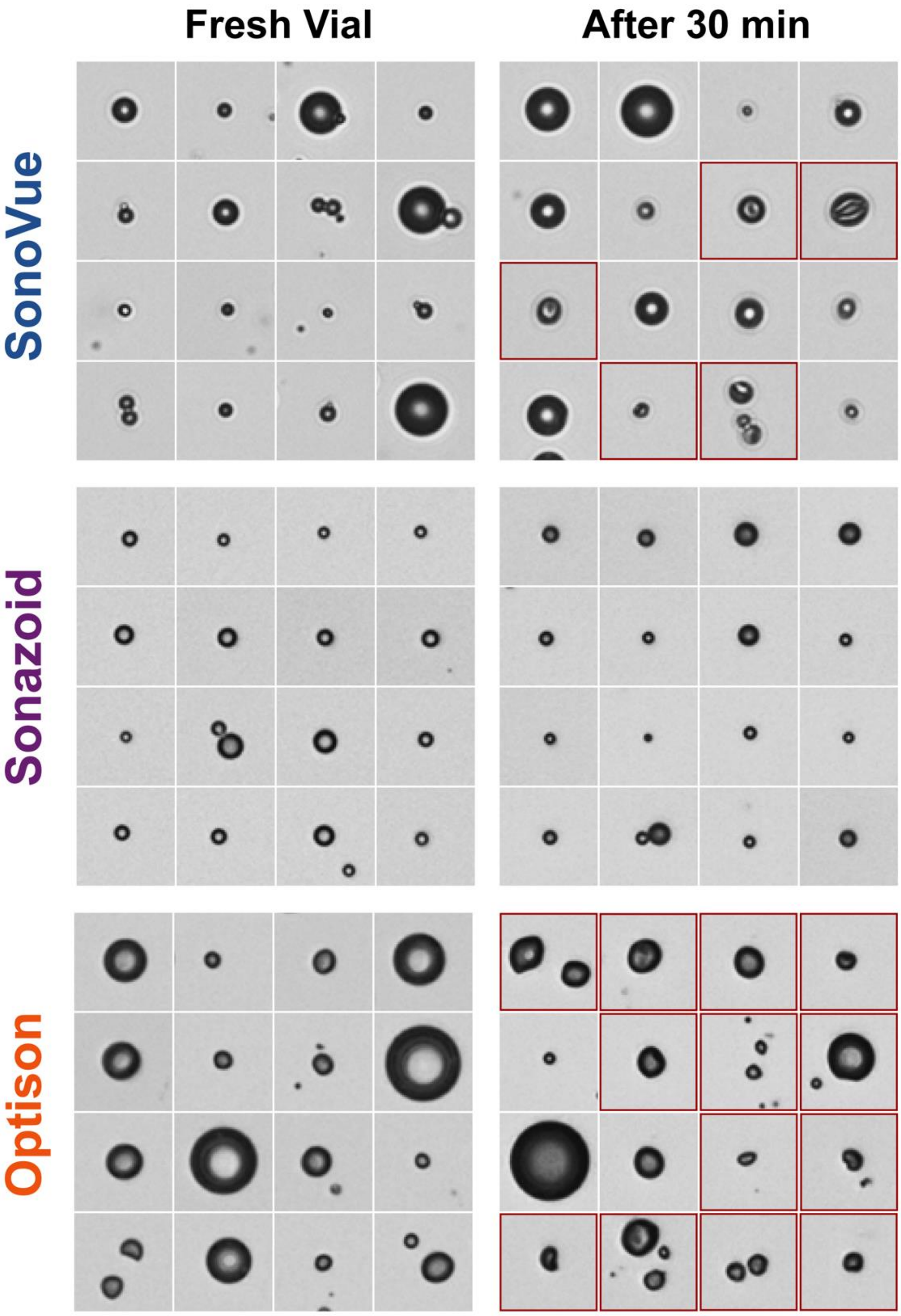

Figure S9. Micrographs of the three microbubble formulations showing the shape of the bubbles when fresh, and after 30 minutes. The boxes outlined in red emphasise the bubbles that are non-spherical, *e.g.*, buckled. After 30 minutes Optison™ exhibited a large portion of non-spherical bubbles.

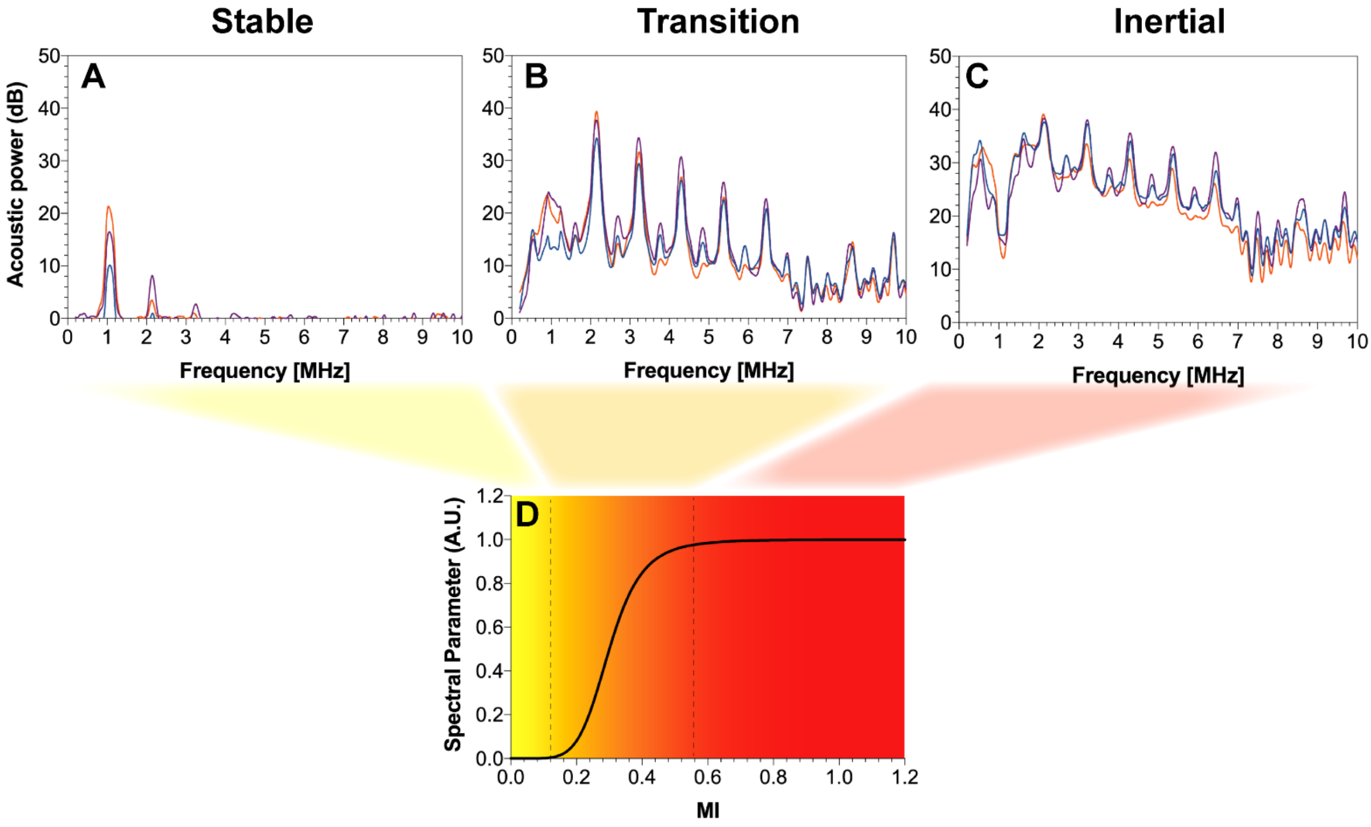

Figure 10. Example frequency spectra obtained from each of the bubbles for the stable (**A**), transition (**B**), and inertial cavitation (**C**) areas in the MI vs. Spectral parameter graphs (**D**). All frequency spectra are taken from the same concentration and MI. An increase in both subharmonic (400–600kHz) and broadband noise was observed.

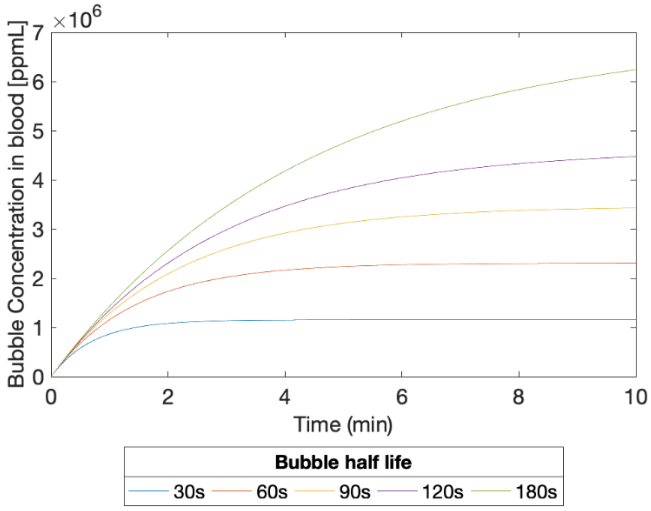

Figure S11. Simulated microbubble blood concentration as a function of time during continuous infusion. A longer half-life resulted in a higher bubble concentration. At the shortest half-life of 30s the peak concentration was 1.5x10^6 ppmL and at the longest half-life of 180s the peak concentration was 6.2×10^6 ppmL.

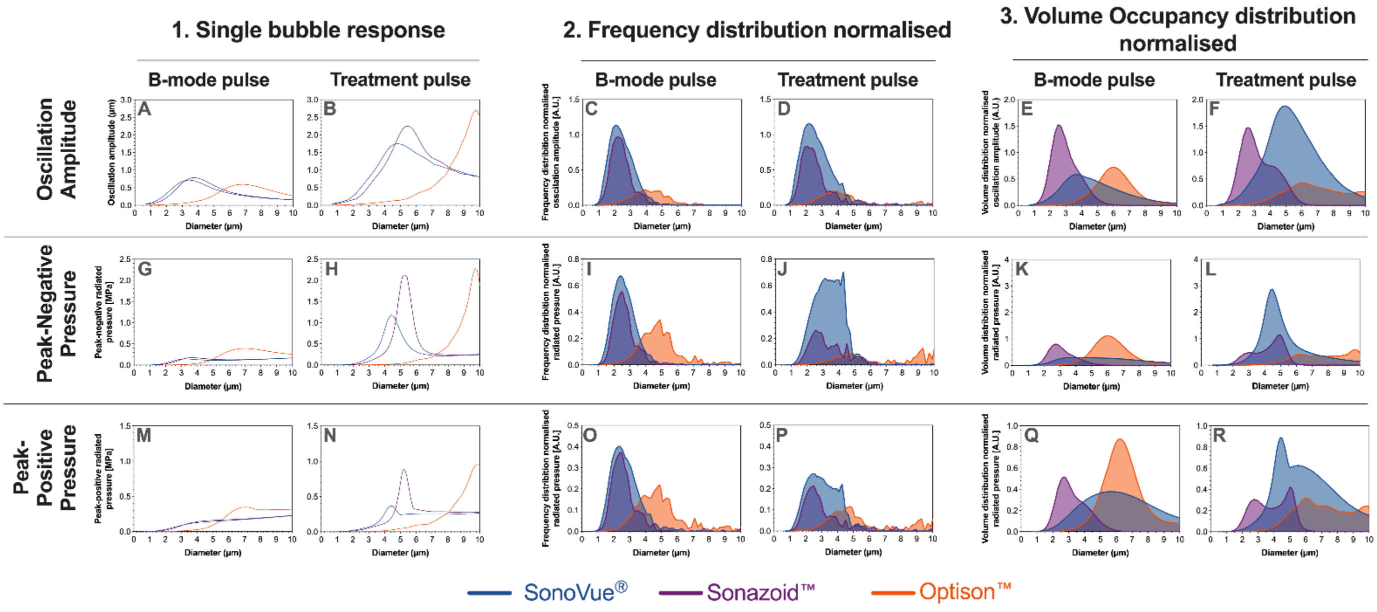

Figure S12. Simulation results showing the oscillation amplitude, peak-negative and -positive radiated pressure induced on a cell 7 μm away from the bubble centre induced from SonoVue®, Sonazoid™, and Optison™. Column 1 shows the raw values for the three parameters evaluated. Column 2 shows the raw values normalised to the frequency distribution, and Column 3 shows the raw values normalised to the volume occupancy distribution. The results are presented for both the B-mode imaging pulse and the long treatment pulse. Normalising to the frequency distribution indicates that SonoVue® would induce the largest overall effect using either the B-mode pulse, or Treatment Pulse. Normalising to the volume occupancy, shows that with the B-mode pulse any microbubble would be most efficient, but with the treatment pulse, SonoVue® would be the most optimal.

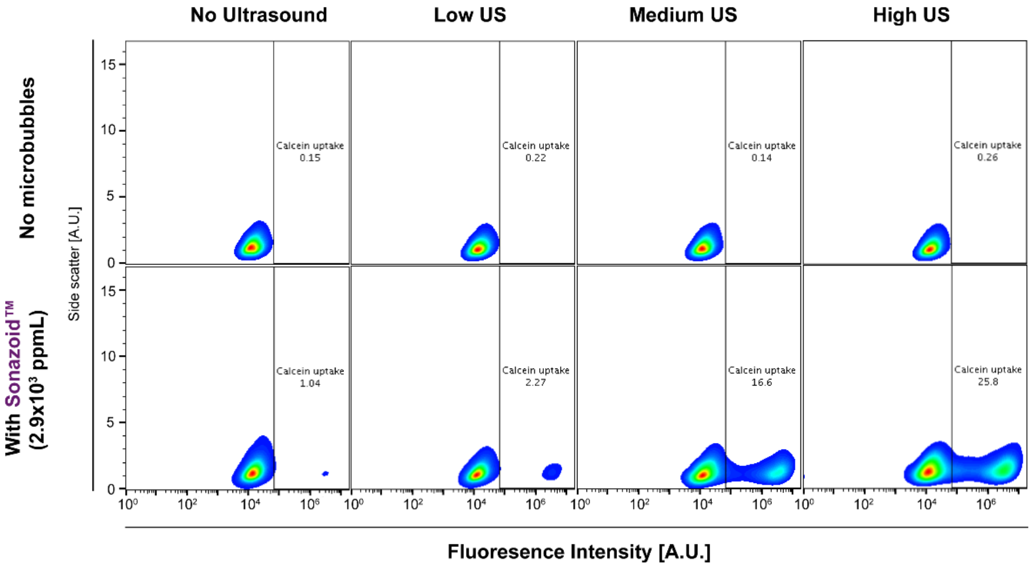

Figure S13. Examples of flow cytometry measurements quantifying the percentage of MIA PaCa-2 cells that were calcein-positive. An increase in calcein-positive cells is seen with increased acoustic power.

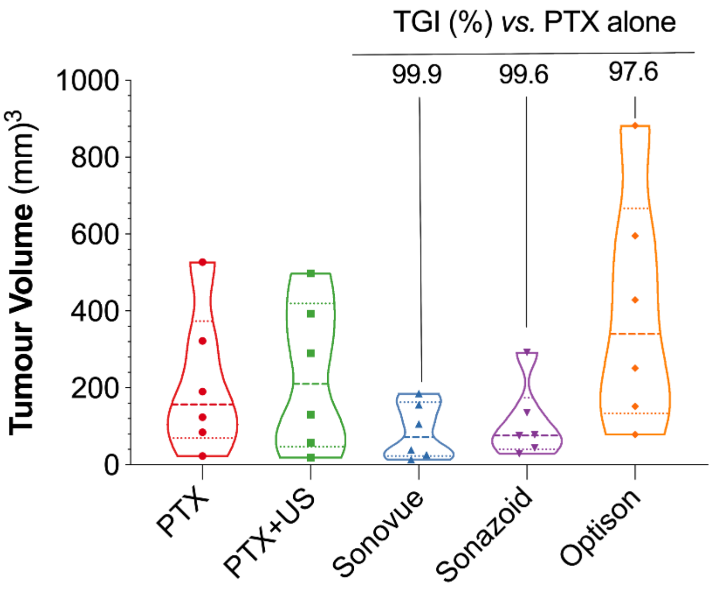

Figure S14. Violin plots show the size distribution of the mice remaining at week 8 with tumour growth inhibition based on median tumour volumes.

Table S1. Parameters selected for inducing sonoporation.

Parameter	Value
**Colour Flow settings**	
Accumulation	1.5
Threshold	0%
Pulse Length (µs)	20
Map Compress	7
Map	V0
Wall filter	0
Frame Average	4
Frequency (MHz)	1.8
Transparency map	None
Packet size	12
Flash Suppression	0
Spatial filter	0
Line Density	4
CF power output (%)	50
B Power output (%)	24
Base line (%)	50
Scale	Maximum
**Contrast Mode settings**	
# Flash MI limit	1.5
# Flash frames	10
SRI HD	2
Suppression	0
Colorize	Tint Contrast 2
Gray Map	Gray Map K
Focus Width	1
Frame Average	2
Rotation	Up
Line Density	4

B Power output (%)	24
Time Delay	1
Dynamic Range (dB)	60

Table S2. On screen parameters using "optimised" sonoporation settings.

**Parameter**	**Value**
**BC**	
MI (MHz)	0.4
TI	0.4
FR	17
Frq	3.0
Gn	24
D	4.0
AO (%)	28
Trig	0-1
Tch	Fnd
fMI	1.3
**CF**	
Frq (MHz)	1.8
Gn	14.0
L/A	4/A
AO (%)	80
PRF	6.4
WF	674
S/P	0/12
fMI	0.5

Table S3. Characteristics of the commercial MBs used in the studies (adopted from the Summary of Product Characteristics (SmPC) approved in Norway unless other references are stated).

**MB formulation**	**Mean Diameter**	**Concentration (MB/ml)**	**In-use storage***	**Recommended dose (adults)**	**Calculated MB-concentration** (MB/ml)**
**SonoVue®** Phospholipid shell	2.5 µm [1]	1-5×10^{8} [1]	6 hours	Max 2.4 ml	0.048–0.24×10^{6}
**Sonazoid™** Phospholipid shell	2.1 µm [2]	12×10^{8} [2]	2 hours	0.015 ml/kg body weight (example 1.2 ml for 80kg)	0.29×10^{6}
**Optison™** Albumin shell	2.5–4.5 µm	5-8×10^{8}	30 min	Normal: 3ml<br>Max: 8.7ml	0.30–0.48×10^{6}<br>0.87–1.39×10^{6}

* SonoVue® and Sonazoid™: after reconstitution; Optison™: after perforation of vial rubber stopper. ** Calculation based on 5 litre blood in the human body, not taking elimination into consideration.

References

1. Schneider, M. Characteristics of SonoVue™. *Echocardiography* **1999**, *16 Pt 2*, 743–746. https://doi.org/10.1111/j.1540-8175.1999.tb00144.x.
2. Sontum, P.C. Physicochemical Characteristics of Sonazoid™, A New Contrast Agent for Ultrasound Imaging. *Ultrasound Med. Biol.* **2008**, *34*, 824–833. https://doi.org/10.1016/j.ultrasmedbio.2007.11.006.
